# Sleep duration and quality trajectories during the early days of the COVID-19 pandemic: a Canadian nationally representative study

**DOI:** 10.1186/s12889-025-22617-3

**Published:** 2025-05-07

**Authors:** Anthony Levasseur, Mathieu Pelletier-Dumas, Éric Lacourse, Jean-Marc Lina, Guido Simonelli, Roxane de la Sablonnière

**Affiliations:** 1 Department of Medicine, Université de Montréal, Montréal, H3T Canada; 2 Centre Intégré Universitaire de Santé et de Services Sociaux du Nord-de-L’Île-de-Montréal, Hôpital du Sacré-Coeur de Montréal, 5400 Gouin O. Blvd, Montréal, QC H4J 1C5 Canada; 3 Department of Psychology, Université de Montréal, Montréal, H3T Canada; 4 Department of Sociology, Université de Montréal, Montréal, H3T 1J4 Canada; 5 Department of Electrical Engineering, École de Technologie Supérieure de Montréal, Montréal, H3C 1K3 Canada; 6 Department of Neuroscience, Université de Montréal, Montréal, H3 T Canada

**Keywords:** Sleep, Longitudinal, Trajectory, COVID-19, Health, Predictor, Correlates, Canada, Mental health, Environment

## Abstract

**Background:**

Poor sleep health has wide-ranging consequences for general health. The year 2020 marked the first year of the COVID-19 pandemic throughout the world, an event that introduced dramatic disruptions to daily life. Studies conducted during the first wave of the pandemic reported a decrease in sleep quality but also an increase in sleep duration, which contradicts the simultaneous decrease in sleep duration reported in Canada. However, prior studies were not representative of the Canadian population. To assess pandemic-induced health disruptions, we investigated sleep health trajectories and health correlates during the first wave of COVID-19 in a longitudinal nationally representative sample of Canadians. We aimed (1) to determine the trajectories of sleep duration and sleep quality, (2) to identify health factors associated with unstable sleep trajectories, and (3) to explore associations between sleep trajectory groups.

**Methods:**

A nationally representative sample of 2,246 individuals residing in Canada was surveyed 6 times between April and July 2020. Participants reported on their sleep and health-related factors (e.g., sociological and demographic factors). We first used latent class growth analysis to identify sleep trajectories. We then used multinomial logistic regression models to determine the relationships between health-related predictors and trajectory groups. Finally, we used joint trajectory analysis to explore the relationships between sleep duration trajectories and sleep quality trajectories.

**Results:**

We identified four constant sleep quality trajectories (6.7%, 37.1%, 45.5%, and 10.7% of the sample). We identified two sleep duration trajectories, one of stable short sleep (33.9% of the sample), and one of long and decreasing (-2.32 min/2 weeks) sleep (66.1% of the sample). Living with someone predicted longer and decreasing sleep duration. Being 25 or older was associated with a lower likelihood of belonging to the long and decreasing sleep duration trajectory. There was a 98.9% likelihood of belonging to the long and decreasing sleep duration trajectory for those belonging to the higher sleep quality trajectory.

**Conclusions:**

In our study, we found no convincing evidence that sleep health indicators deteriorated during the first wave of COVID-19 in Canada. The overall stability of sleep suggests that sleep is likely governed by factors that remained stable.

**Supplementary Information:**

The online version contains supplementary material available at 10.1186/s12889-025-22617-3.

## Background

Poor sleep health has far-reaching and wide-ranging consequences for physical and mental health. According to a 2022 umbrella review of 85 meta-analyses, highly suggestive evidence supports the association between long sleep duration and an increased risk of all-cause mortality, and suggestive evidence supports the association between short sleep duration and an increased risk of overweight and/or obesity, as well as poor sleep quality and an increased risk of diabetes mellitus [1]. Poor sleep quality has alsobeen causally related to mental health difficulties [2]. Insomnia has been identified as a predictor of suicidal thoughts and behaviors [3, 4], anxiety [5, 6], and later onset of psychopathologies such as depression and psychosis [7, 8]. Short (< 7 h/night) and long (> 9 h/night) sleep durations are also associated with a higher risk of cognitive disorders [9]. Disrupted sleep negatively impacts mental health in adults [10], and poor sleep quality is causally related to the experience of mental health difficulties [2]. In Canada, short sleep and poor sleep quality are highly prevalent, with approximately one-quarter of Canadian adults reporting not receiving sufficient sleep and up to one-fifth not finding their sleep “refreshing” [11].

The year 2020 marked the first year of the COVID-19 pandemic, an event that introduced dramatic social changes [12] into daily life throughout the world. Many studies have reported an increase in the prevalence of sleep problems around the globe during the COVID-19 pandemic between the prepandemic period, generally referred to as before January 30 2020, and June 2020. These studies reported a decrease in sleep quality [13–15], an increase in sleep duration [14, 16, 17], and an increase in insomnia symptoms [18, 19]. However, there was no consensus regarding what period corresponds to “prepandemic” sleep, as many studies invited participants to set their own reference for prepandemic sleep which may not correspond to the same period throughout their sample. This may encourage participants to compare their current sleep with the period they associate with having the best sleep, even if it was impacted by the pandemic in some way, thus failing to measure the pandemic’s effects on sleep. The aforementioned studies also used retrospective measures of prepandemic sleep, exposing themselves to recall bias. Participants might be inclined to perceive their prepandemic or early-pandemic sleep favorably, as they might believe that their sleep was impacted during the pandemic by the measures in place. A longitudinal study by French et al. [20] measuring sleep quality both in late March 2020 and a month later also reported a decrease in sleep quality between these time points. However, it relied on a retrospective measure of sleep quality ("in the last 3 months"), which is susceptible to recall bias. It is suggested that these changes were at least partially driven by the widespread COVID-19-related disruptions of daily life, such as home confinement, changes in work and social habits, and threats to health caused by the virus [21, 22]. In Canada, during the first wave of the pandemic (broadly defined as the period between late January to the end of June or mid-July of 2020 [23, 24]), a handful of studies reported on sleep health outcomes, reflecting a sleep quality decrease as well, but contradictory results regarding changes in sleep duration. A retrospective increase in the emergence of sleep difficulties and an average decrease in sleep duration was reported by Robillard et al. during the first COVID-19 wave (i.e. in the 7 days before filling out the survey) in comparison with preoutbreak times (i.e. in the last month before the outbreak). [25] A retrospective decrease in sleep duration between the start of the COVID-19 pandemic (“since COVID-19”) and April–May 2020 was also reported by Carroll et al. in 34% of their adult participants. [26] Morin et al. also reported a decrease in sleep quality and an increase in insomnia between 2017–2018 and April–May 2020. [19] However, none of these studies were representative of the Canadian population, as Robillard et al.’s study included a large proportion of white, highly educated, high-income, highly employed, middle-aged women; Carroll et al.’s study was limited to parents of families from the province of Ontario; and Morin et al.’s study was limited to French-speaking Canadians, residing mostly in the province of Québec. The samples in Robillard et al.'s and Carroll et al.'s studies may be characterized by greater resource availability, which could enable better coping with the negative impacts of crises. This may mitigate mental health and sleep health issues, potentially limiting the generalizability of their findings. Morin et al.’s sample was predominantly from Québec, a region in which additional COVID-19 policies were implemented, in comparison to the rest of Canada, such as closures of non-essential businesses and regional travel restrictions, [27, 28] likely increasing the burden of the pandemic on daily life. To evaluate the impact of the COVID-19 pandemic on Canada's population, it is essential to gather a sample that reflects its demographics in terms of age, gender, and province of residence, thereby enhancing the representativeness of the findings. To achieve this, our study will choose participants according to established quotas for these three sociological and demographic variables and apply weighting adjustments to address any identifiable sociological and demographic discrepancies in our sample. The use of different questionnaire items may also explain the discrepancies between prior Canadian and foreign studies, as nearly half of the aforementioned studies used researcher-developed tools or device-based measures.

During the early days of the pandemic, several studies attempted to identify factors associated with sleep disruption. Studies conducted worldwide have revealed associations between poorer sleep health and various sociological, demographic, environmental, biological, and subjective exposures to COVID-19 factors, such as younger age [20, 29–34], identifying as a woman [30, 34–39], low social support [19], living with children [25, 39–41], being isolated or quarantined [42, 19, 33, 38, 43], being exposed to a high COVID-19 threat [40, 44], working in healthcare [39, 40, 45], being concerned with COVID-19 [30, 33, 34, 46], being diagnosed with COVID-19 [40, 43, 47], and experiencing financial difficulties [19, 33, 39, 40]. Being a racialized minority (e.g. being a member of a Black or Asian ethnic group) in the United States of America and in the United Kingdom was also associated with being more vulnerable to sleep health disparities during the first wave of the COVID-19 pandemic [39, 48]. In Canada, visible ethnic minority groups are defined as “'persons, other than Aboriginal peoples, who are non-Caucasian in race or non-white in color” [49]. However, it was reported that Aboriginal peoples were eight times more likely to die from COVID-19 compared to non-aboriginal individuals in Canada [50], with high levels of overcrowding, inadequate housing, and limited access to healthcare services documented as significant factors contributing to the increased risk of transmission and severe illness [51]. This suggests that belonging to an ethnic minority group, or to an Aboriginal people, may predict worse sleep in Canada.

However, an important limitation of this body of work is that most of these studies had cross-sectional designs, used retrospective data, and used sleep items that referred to different periods as “prepandemic”. Accordingly, cross-sectional sleep data may not capture lifestyle changes impacting individuals, particularly in the context of a rapidly evolving environment and fast-changing policies that may include income support, border control, travel, transport, and quarantine measures [52]. In contrast, a longitudinal assessment of sleep during the early days of the pandemic could identify changes in sleep (i.e. unstable sleep), that may have been pandemic-induced disruption. To date, no published study has surveyed sleep duration and quality multiple times during the early days of the COVID-19 pandemic and explored predictors of sleep health outcomes in a representative sample of Canadians.

Sleep duration and sleep quality are key concepts of sleep health [53]. Sleep duration and quality are both distinct in that they can impact health differently but are also inextricably linked, as individuals with short and long sleep durations are also those most likely to report sleep disturbances [54], which is indicative of poor sleep quality. The evidence suggests that the effects of sleep duration and sleep quality on health outcomes are also not simply additive, calling for measurements of both aspects of sleep when possible [54]. This evidence highlights the need to determine the interplay, if any, between sleep duration and sleep quality.

To address the limitations outlined above, we present the first longitudinal investigation of sleep trajectories and their relationships with COVID-19-related factors in a representative sample of Canadians during the first wave of the COVID-19 pandemic. The present study aims to (a) determine sleep quality and sleep duration trajectories in our sample during the first COVID- 9 wave; (b) identify COVID-19-related sociological, demographic, environmental, biological, and other exposure predictors of unstable sleep quality and duration trajectories; and (c) determine associations between sleep quality and sleep duration trajectories. Determining sleep trajectories during the first wave of COVID-19 would allow for a better understanding of the immediate impact of a public health crisis of this scale on sleep and an assessment of its evolution. A better understanding of the predictors of sleep health trajectories and associated sleep trajectories could be used to develop better sleep programs to mitigate the impact of public health crises comparable to COVID-19 on sleep health. For instance, another viral outbreak of the same scale as COVID19 in the future could lead to home confinement measures once again. Knowing which populations are most vulnerable to the effects of home confinement on sleep health in advance may encourage policy adjustments for these populations. We hypothesized that (1) sleep quality and sleep duration were both unstable in at least one trajectory group per outcome variable and that (2) younger age, identified as a woman, living alone or with minors, reporting a higher local COVID-19 spread speed, staying at home frequently, being in voluntary isolation, being employed in healthcare, being very concerned about getting very sick with the virus, being concerned about peers getting very sick with the virus, having been diagnosed previously with the virus, having peers that have been diagnosed previously with the virus, being concerned about the financial impact of COVID-19 on oneself, reporting any impact of the crisis upon one’s personal life, following the government’s COVID-19 recommendations, identifying to a visible minority group of Canada (African-American, Latino or Hispanic, Asian, or other than White/Caucasian) or to an Aboriginal People significantly predicts unstable sleep quality and sleep duration trajectory group membership, and that (3) belonging to an unstable sleep duration trajectory group predicted belonging to an unstable sleep quality trajectory and vice-versa.

## Participants and methods

### Study setting

This study is part of a larger ongoing study that seeks to comprehend the overall impacts of the COVID-19 pandemic on the Canadian population [55]. The goals of the COVID-19 Canada Survey are to be better prepared for a potential subsequent public health crisis and to maximize the overall resilience of our society. This study makes use of 6 measurements spread over 14 weeks. This study provides an interim analysis of sleep duration and quality data collected during the first measurement times. The IRB approval for this study was given by the Research Ethics Committee in Education and Psychology of the Université de Montréal [55].

### Data collection

**Fig. 1:**
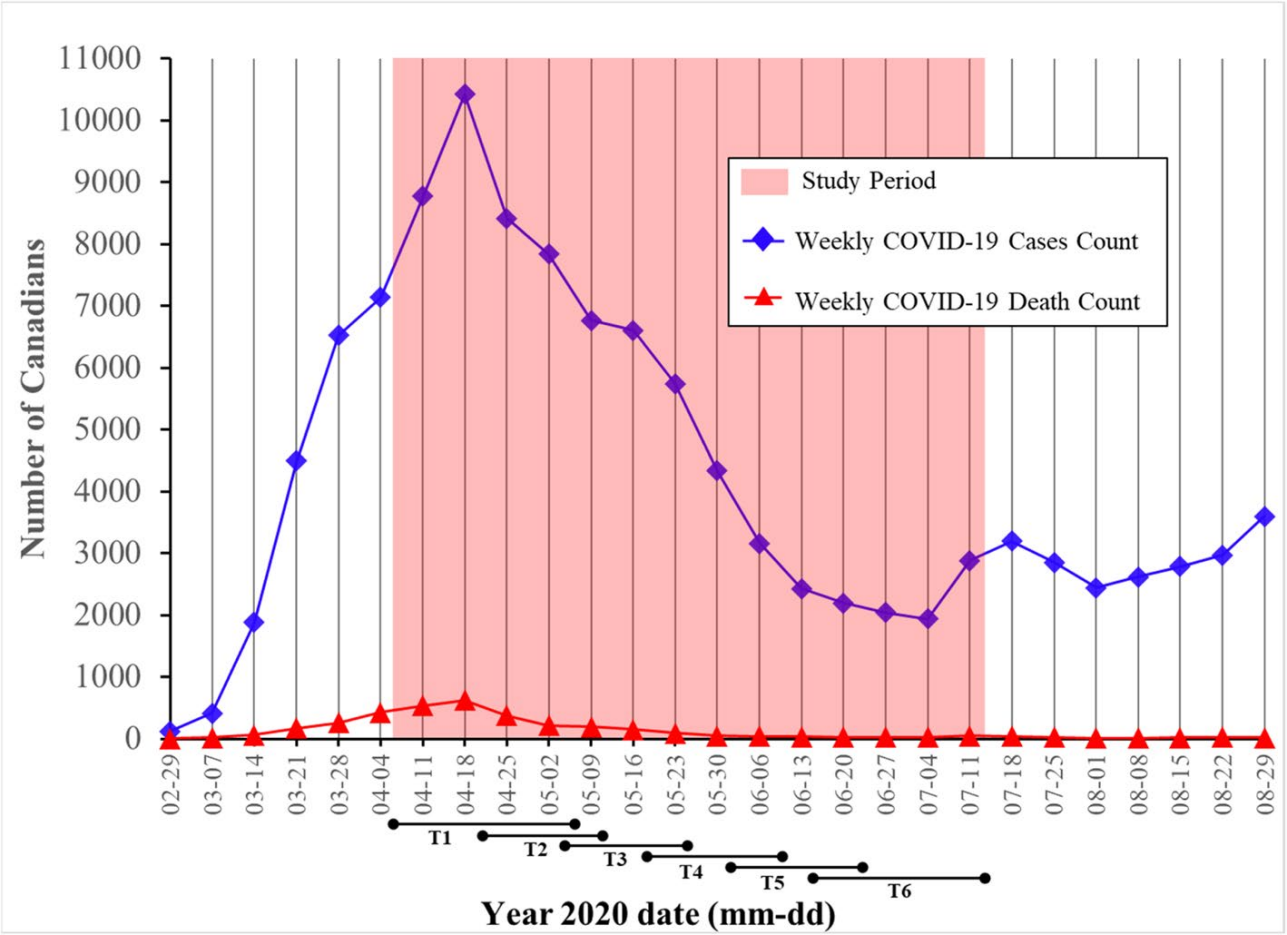
Weekly COVID-19 cases and death count in Canada. *Note*. Figure depicting the evolution of weekly COVID-19 cases and death count in Canada during the first measurement time of the COVID-19 pandemic, with the study period highlighted in red, and study assessment periods underlined under the graph. T1 – T6: Study measurement time 1 to 6. Figure built based on data collected from the *Public Health Agency of Canada* [62].

**Fig. 2:**
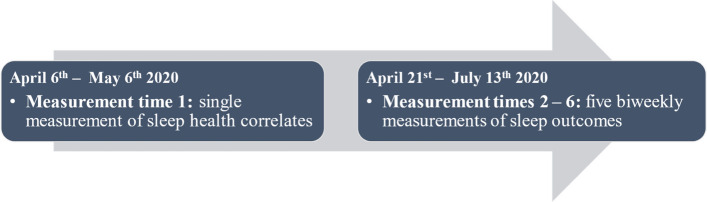
Survey timeline. *Note*. Illustration of the series of measurements performed to gather the dataset utilized in this study, along with the corresponding time duration for each measurement time point

This longitudinal study on Canadians was conducted during the first wave of the pandemic (see Fig. 1) [23, 24]. To capture the effects of COVID-19 on sleep during the first wave, we aimed to collect data during the peak of infection. To that end, we collected data between April 6 and July 13 of 2020 (see Fig. 1). Data was collected through the *AskingCanadians* (French: *Qu’en pensez-vous*) web panel from the survey firm Delvinia [56] which recruited Canadian adults. The firm used SMS messaging and mobile app reminders to reduce attrition and was carried out online in French and English. Delvinia selected participants based on established quotas from the 2016 Census Profile [57] of Statistics Canada for three sociological and demographic variables: age, gender identity, and province of residence (for details, see COVID-19 Canada’s Technical Report [55]). In the survey, eligible participants were invited to complete a questionnaire measuring sleep health predictors once during the first measurement time and to complete a questionnaire measuring sleep outcomes 5 subsequent times (see Fig. 2). Each survey given to participants included a welcome paragraph along with a consent form. Informed consent was given by all participants prior to completing each survey. Participants received a compensation of approximately 2.50 Canadian dollars per completed questionnaire, with the payment provided as points that could be redeemed at partner companies of their choice. The survey used a rolling cross-sectional survey design [58]. During the first measurement time, the survey was made available to a large pool of participants, which was divided into 14 subgroups. During this time, on each day over a 14-day span, one subgroup from this pool was invited to complete the questionnaire until approximately 250 surveys were filled. For subsequent measurement times, participants who completed the survey during the first measurement time were invited to participate again. These participants were contacted in the same order as the initial round. Each subgroup received their next invitation 14 days after their previous one. For each measurement time, participants had between 7 and 14 days to fill the questionnaire (see Table 1). The completion time per questionnaire ranged from 15 to 20 min. Participants who missed one or more waves were able to participate again at any subsequent measurement time. A procedure of planned missingness was implemented across many variables including three of our predictor variables but not our outcome (sleep) variables [59, 60]. The three predictors were “Concern for self getting very sick with the virus”, “Concern for a peer getting very sick with the virus”, and “Concern about the financial impact of COVID-19 on self”. Variables under planned missingness were presented to 66% of the sample. According to Enders [61], the utilization of planned missingness allows for the maintenance of validity and statistical power without any compromise. This procedure consists of the presentation of questionnaire items only to subsets of participants to reduce the length and cost of the data collection process [59]. Therefore, the questionnaire items were not always presented to the same participant from one measurement time to the other. The predictors of sleep health were measured only once at measurement time 1, before our outcome variables. The sleep variables were measured 5 times, at measurement times 2 to 6. To mitigate the potential impact of question position contamination, the questions were organized into 10 blocks, each addressing specific COVID-19 issues, and were administered in a randomized order [55].

**Table 1:** Study population characteristics

Measurement Time	Missing Data (%)	Sample Size (*N*)	% (*n*) Women	Mean Age (range)	Survey Date^a^ (year 2020)	No. of Days to Complete the Survey
1	0	2246	49.5 (1111)	49.85 (68)	April 6 – May 6	14
2	16.4	1878	41.0 (921)	50.39 (68)	April 21 – May 11	7
3	12.2	1974	43.4 (974)	49.93 (68)	May 4 – May 25	7
4	11.6	1987	42.9 (963)	49.84 (68)	May 18 – June 10	7
5	15.9	1891	40.8 (916)	50.28 (68)	June 1 – June 23	7
6	16.5	1878	41.5 (933)	50.30 (68)	June 15 – July 13	14

*Note*. ^a^includes the number of days to complete the survey

### Participants

#### Inclusion and exclusion criteria

To be eligible, participants were required to be at least 18 years old and to be Canadian citizens or permanent residents. A total of 3,617 Canadians were recruited at the first measurement time of the survey.

To be included in our analysis, participants had to have answered both sleep duration and sleep quality questions at least three times each (minimum number of measurement times required to model a linear trajectory of change) from measurement time 2 to 6. Responses provided in less than 4 min and those associated with the failure of the two attention check items included within each survey during measurement times 2 to 6 were considered invalid, and they were excluded from the sample. Of the total number of responses, 3.7% and 0.2% were excluded due to the failure of both attention check items and the completion of a questionnaire in 4 min or less, respectively. Furthermore, we excluded 1,371 participants (37.9%) who did not answer questions at least three times regarding their sleep duration and/or quality, leaving 2,246 participants as the final sample.

#### Representativeness of the sample

On the first measurement time, the sample reflected the adult Canadian population as described by Statistics Canada’s 2016 census profile data [57] in terms of age, gender identity, and province of residence. The sample was also comparable based on variables that were not included in the quotas such as ethnicity (as shown by Table 3), as well as household size, current employment status, and country of origin [55]. While the full information maximum likelihood is utilized in our trajectory analysis, missing data caused by attrition can have a significant effect on both the results and the representativeness of the sample. Accordingly, to reduce the differences between our sample and the population of Canada, we employed a raking weighting method [63] to correct for identifiable sociological and demographic variations in our sample. To identify sociological and demographic variations, we utilized data from 2016 and 2020 from Statistics Canada [57, 64]. Table 3 shows that our sample also reflected the population of Canada in terms of ethnic background, as none of the Phi coefficients exceed a value of 0.19, indicating negligible associations between the presence of the ethnic background and the data source (0 = study sample, and 1 = Census data). We performed weighting based on the following benchmark variables: 1) presence of household members under 18 years old, 2) province of residence, and 3) Aboriginal background (Canadian Indigenous community members). The weighting procedure was conducted under the function “calibration” from the *Icarus* package in R software. The weighting process had a maximum weight range of 9.56, which enabled the creation of weights ranging from 0.5 to 10.06, with a mean weight of 1. The weighting process led to an 11.30% reduction in bias, according to the selected benchmark variables.

### Measures

#### Sleep outcomes

Our outcome variables, sleep duration and sleep quality, were measured 5 times from April 21 to July 13, 2020. The participants were given a “Prefer not to answer” option every time.

According to the National Sleep Foundation [65], to capture sleep quality, participants can rate their sleep as good, bad, or something in between. Thus, we assessed sleep quality with the question: “*How would you describe the quality of your sleep during the last 24 h?*”. To capture a wide spectrum of sleep quality ratings, the participants answered a Likert scale ranging from 1 (“*slept very badly*”) to 10 (“*slept very well*”). Participants had to answer recall only the last 24 h to mitigate recall bias.

The sleep duration item used similar phrasing to the validated Pittsburgh Sleep Quality Index [66]. Whereas the PSQI measures sleep duration with the question"*During the past month, how many hours of actual sleep did you get at night*", our item assessed sleep duration with the question: “*How much sleep did you get in the last 24 h? (please insert the possibility for hours and minutes)*”. Participants entered the number of hours and minutes they had been asleep during the last 24 h. Sleep duration was then converted to minutes.

#### COVID-19-related predictors of sleep health

Our predictors consisted of sociological, demographic, environmental, biological, and other exposure factors to the virus. Table S1 in Additional File A1 details all predictors and their origins.

The “healthcare-related employment” predictor was determined based on the answer provided to the question, “*What is your current job or profession?*”. The responses were evaluated and categorized using Canada's National Occupational Classification, which is the standardized system for describing occupations in the country [67]. Subsequently, dummy coding was utilized to indicate whether the provided answer corresponded to a “health occupations” category.

### Statistical analyses

All analyses were conducted using SAS version 9.4 software and the PROC TRAJ procedure [68, 69]. Statistical significance was set at a bilateral alpha level of 0.05*.* We utilized Nagin's [70] semiparametric group-based modeling approach to fulfill the first objective and performed a latent class growth analysis with an intraclass variance fixed to zero. The growth model was based on a censored-normal distribution [70], and time was coded in weeks, from 0 to 8. More specifically, the first measurement of sleep, which spanned 2 weeks (April 21 – May 3), with an additional week for late responses (meaning that it could be answered on May 11 at the latest for those meant to answer on May 3), was coded as 0. The second measurement of sleep, which started two weeks after the intended response time for measurement time 1, was coded as 2, and so on in increments of 2, until the final measurement time, coded as 8. First, we determined the optimal number of trajectory groups from one to five third-order trajectory groups by applying the requirement of a minimum of at least 5% of the sample (*n *= 112) per group [71]. Second, we determined the optimal order for each trajectory group based on the Bayesian Information Criterion (BIC), keeping the model that had a value closest to zero [70, 72]. Given the impossibility of specifying to the PROC TRAJ SAS package the real limits (between 1 and 10) of our distribution of sleep quality, all estimated trajectory parameters exceeding the maximum of 10 were adjusted to 10, and all values inferior to 1 were adjusted to 1. We defined ‘unstable sleep trajectory’ as a trajectory identified by latent class growth analysis with at least one statistically significant coefficient of a greater order than 0.

We performed a Pearson correlation matrix of all predictors except age and gender to reject multicollinear predictors that met or exceeded an absolute correlation coefficient value of 0.3 with each other. We performed multinomial logistic regressions using the “RISK” function to estimate the likelihood of being assigned to specific trajectory groups in comparison to the first trajectory group (i.e., the lowest sleep quality group or shortest sleep duration group) as a baseline based on individual-level factors (i.e., our COVID- 9-related sleep health predictors). We computed 3 models per outcome variable: the first model utilized age groups and gender identity only as predictors, using the youngest age category (18–25 years old) as the baseline category, the second model utilized all other valid predictors except ethnic categories, and the third model utilized only “Non-White Ethnicity” as predictor, a binary variable representing participants that did not report “White/Caucasian” as one of their ethnic backgrounds. Participants that selected “Prefer not to answer” to answer the ethnicity item were treated as missing data and were not included in the “Non-White Ethnicity” category. This variable was created to account for the small number of cases of Non-White ethnic backgrounds and to avoid the use of multicollinear predictors. We categorized the variable “Age” in 10-year increments. The variables related to household size were dummy coded; “Plural household size” described participants who lived with someone, whereas “Presence of household members below [age]” described participants who lived with minors under 18 or 6 years of age.

Finally, we used the joint trajectory analysis approach created by Jones et al. [68] to build two models to test the associations between the trajectory groups of our two outcome variables.

#### Missing data strategy

Missing data were assumed and treated as missing completely at random (MCAR) for predictors with planned missingness. Missing data was handled with the full information maximum likelihood (FIML) method, allowing us to include participants with missing data without replacing missing values on the variables used to create the trajectory groups [59, 68, 70]. FIML is currently considered a best practice method for dealing with missing data, regardless of the missingness mechanism [61]. However, no estimation was performed for missing data for other predictor variables because they either represented very small proportions of our sample [see Additional File A1 Tables S6 - 9] or did not meet all validity criteria for analysis. Furthermore, we treated “healthcare-related employment” predictor responses as missing data for answers that could not be categorized via Canada’s National Occupation Classification system. Treated as missing data were also “Prefer not to answer” responses.

## Results

### Sample characteristics

At measurement time 1, participants in the final sample were aged between 18 and 86 years old (*M* = 49.85; *SD* = 16.76), and the female gender identity represented 49.5% of participants (*n* = 1111; with 0% “other”) [see Additional File A1 Tables S6 and S8]. The distribution of participants by province of residence is displayed in Table 2. The ethnic distribution of our sample, along with Statistics Canada’s 2016 Census data, is displayed in Table 3.

**Table 2:** Sample distribution according to the territory of residence

Measurement time	Maritimes^a^		British Columbia		Ontario		Québec		Prairies^b^		Newfoundland and Labrador	
	*n*	%	*n*	%	*n*	%	*n*	%	*n*	%	*n*	%
1	135	6.0	320	14.2	919	40.9	439	19.5	403	17.9	30	1.3
2	122	5.4	276	12.3	777	34.6	335	14.9	340	15.1	28	1.2
3	111	4.9	286	12.7	809	36.0	395	17.6	347	15.4	26	1.2
4	119	5.3	296	13.2	811	36.1	385	17.1	350	15.6	26	1.2
5	124	5.5	274	12.2	793	35.3	321	14.3	355	15.8	24	1.1
6	117	5.2	259	11.5	786	35.0	358	15.9	332	14.8	26	1.2

*Note*. ^a^New Brunswick, Nova Scotia, and Prince Edward Island. ^b^ Alberta, Manitoba, and Saskatchewan

**Table 3:** Sample and census ethnic background distribution

Ethnic Background	Sample data (*N* = 2,246)		Canadian Census Data (2016) [57]	Pearson Chi-Square Goodness of Fit		Phi Coefficient^a^
	*n*	%	%	χ^2^ (1)	*p*	φ
White/Caucasian	1393	62.0	72.9	11.93	.001	.08
African-American	23	1.0	3.5	23.65	<.001	− .07
Latino or Hispanic	22	1.0	1.3	0.02	.892	0.00
Asian	237	10.6	15.0	2.26	.133	− .02
Aboriginal	23	1.0	4.9	45.56	<.001	− .10
Other/Unknown	61	2.7	2.4	8.81	0.003	0.03
Prefer not to say	31	1.4	NA	NA	NA	NA
Missing	504	22.4	NA	NA	NA	NA

*Note*. ^a^Association between the presence of the ethnic background and the data source (0 = study sample and 1 = Census data)

### Sleep behavior during the first wave of the COVID-19 pandemic

The mean sleep duration within 24 h was 442.76 min (*SD* = 75.70), while the mean sleep quality score was 6.87 (*SD* = 1.89). The distribution of sleep quality was negatively skewed, with less than 22% of participants reporting a sleep quality of 5 or less [see Additional File A1 figure S4], reflecting Statistics Canada’s reported 1 in 5 adults not finding their sleep refreshing. All values of sleep duration were plausible, although they exceeded Statistics Canada’s reported value of 1 in 3–4 adults with insufficient (7–9 h/night) sleep [11], with 58.4% of adults getting less than 7 h or more than 9 h of sleep on average. No outliers were detected. Sleep duration and sleep quality were significantly associated, with higher sleep quality being associated with longer sleep duration (*r* = .464, *p* < .001). Additional descriptive characteristics of the outcome variables are available in Additional File A1.

### Trajectories of sleep duration and sleep quality during the first wave of the COVID-19 pandemic

**Fig. 3:**
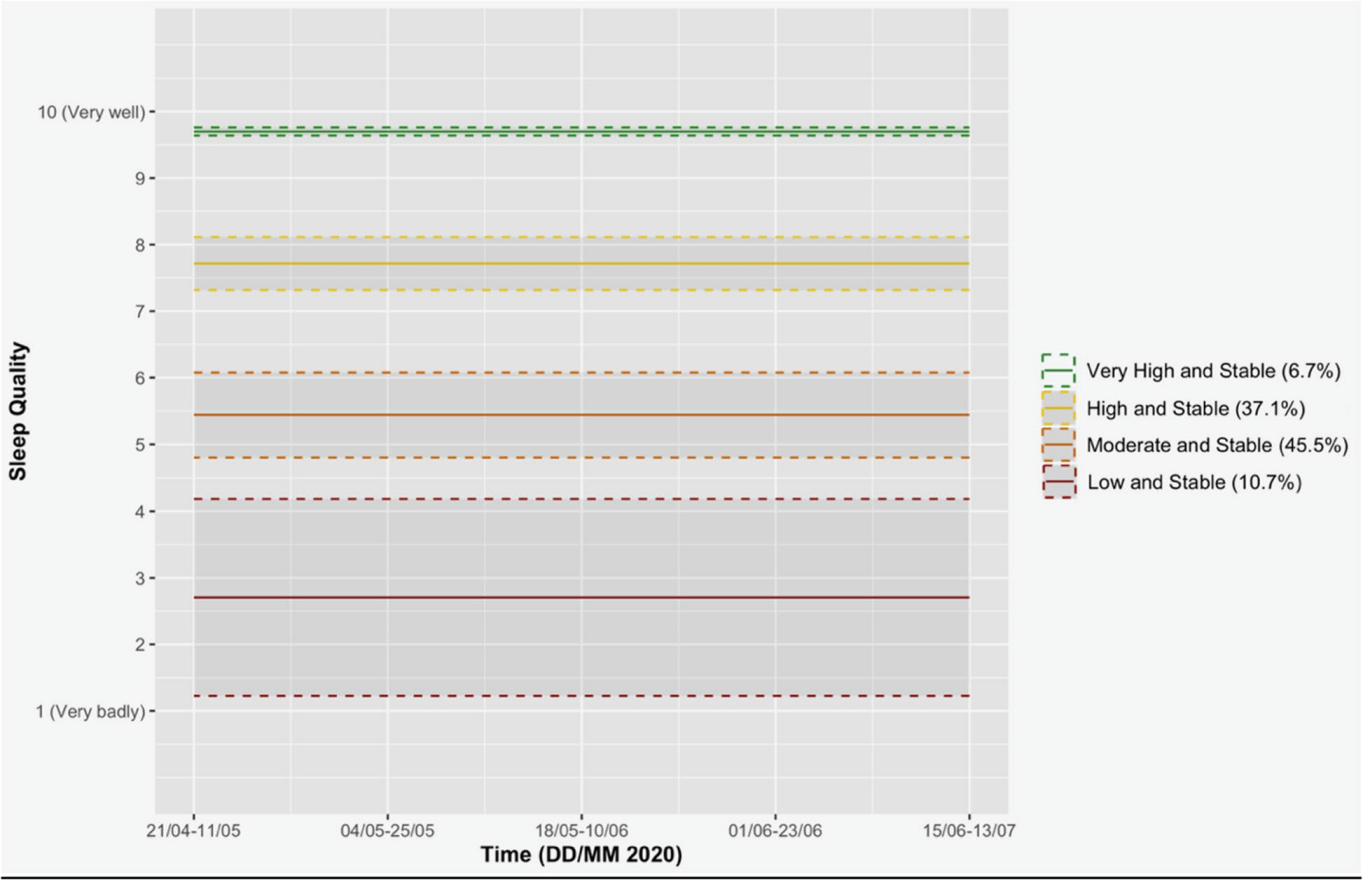
Selected sleep quality model. *Note*. Figure illustrating the selected sleep quality trajectory model across the 5 measurement times

We identified four trajectory groups of sleep quality, and 0 as the optimal order for each trajectory, meaning that the 4 trajectory groups were best described by constants. The chosen model (see Table 4 and Fig. 3) shows that sleep quality remained constant and that participants reported, on average, a quality of 2.48 (6.7% of the sample), 5.44 (37.1% of the sample), 7.83 (45.5% of the sample) and 10 (10.7% of the sample) out of 10.

**Table 4:** Coefficient estimates for the group-based trajectory model of sleep quality

Trajectory Group	Estimate	*SE*
1. Low and stable	2.48*** (intercept)	0.60
2. Moderate and stable	5.44*** (intercept)	0.27
3. High and stable	7.83*** (intercept)	0.15
4. Very high and stable	10.00*** (intercept)	0.22

*Note.* ***p < .001e- 3; *SE* = Standard Error

**Fig. 4:**
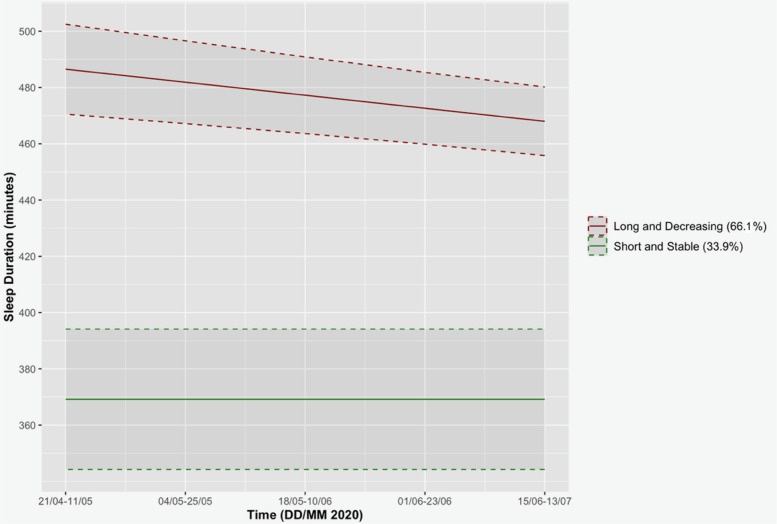
Selected sleep duration model. *Note*. Figure illustrating the selected sleep duration trajectory model across the 5 measurement times

We identified 2 trajectory groups of sleep duration and optimal orders of 0 for the shortest duration trajectory and 1 for the longest duration trajectory. The chosen model (see Table 5 and Fig. 4) suggests that 33.9% of the participants had a shorter sleep duration that averaged 369.18 min and remained constant, whereas the other 66.1% had a longer sleep duration of 486.53 min, which initially decreased linearly at a rate of 2.32 min per week.

**Table 5:** Coefficient estimates for the group-based trajectory model of sleep duration

Trajectory Group	Parameters	Estimate	*SE*
1. Short and stable	Intercept	369.18*** (intercept)	12.73
2. Long and decreasing	Intercept	486.53*** (intercept)	8.16
	Linear	− 2.32***	0.52

*Note.* ***p < .001; *SE* = Standard Error

Additional File A1 Table S11 displays the Bayesian Information Criterion (BIC) values and group sizes for model selection based on the number of trajectory groups and the order of the trajectories of the model with the optimal number of trajectory groups. Figures S1 and S2 in the Additional File show the individual trajectory models for all participants for each trajectory group identified, labeled by trajectory number. 

### Associations between sleep trajectory belonging and COVID-19-related predictors of sleep health during the first wave of the COVID-19 pandemic

The predictors “healthcare-related employment” and “Self has been diagnosed with the virus previously” were removed from further analyses, as less than 5% of the participants answered each question positively (3.1% and 0.2%, respectively), thus leading to a lack of statistical power to compute the logistic regression model.

The Pearson correlation matrix (see Additional File A1 Table S10) identified five correlation values exceeding an absolute value of 0.3, leading to the rejection of “Concern for a peer getting very sick with the virus”, which was positively correlated (*r* = .62, *p* < .001) with “Concern for self getting very sick with the virus”, and “frequency of stay at home”, which was positively correlated (*r* = 0.45, *p* < .001) with “Self is following the government’s recommendations”. No action was taken for the correlation between Presence of Household Members Under 18 and Age group 35 to 45, as it was considered to be acceptable in magnitude (*r* = .30, *p* < .001). No action was taken for the correlations between ethnic category predictors as well, as they were replaced with the “Non-white” predictor. This intervention supports that the absence of statistical significance in individual predictors cannot be attributed to overlapping predictors.

To explore the relationship between predictors and trajectory group membership, a total of 6 logistic regression models were computed (for full tables, see Additional File A1 Tables S12 - 17), accounting for a maximum of 1 predictor per 15 participants belonging to the smallest outcome category. Few statistically significant associations were found. For sleep duration trajectories, in the age and gender model, we found that participants who reported being 25 years or older were consistently less likely to belong to the long and decreasing (Age group 25–34: *OR*] = 0.35; 95%*CI* = 0.18–0.70; *p* = .003, age group 35–44 *OR* = 0.30; 95%*CI* = 0.15–0.60; *p* = .00 1, age group 45–54 *OR* = 0.28; 95%*CI* = 0.14–0.53; *p* < .001, age groups 55–64 [*OR* = 0.34; 95%*CI* = 0.19–0.63; *p* < .001, age group 65 + *OR* = 0.41; 95%*CI* = 0.22–0.76; *p* = .004) sleep duration trajectory group than participants who reported being 18 to 25 years old. In the model containing all other predictors, participants who reported a household size greater than one were 2.57 times more likely to belong to the long and decreasing sleep duration trajectory group (*OR* = 2.57; 95%*CI *= 1.43–4.60; *p* = .002), than participants who reported a household size of one. Regarding sleep quality trajectories, in the age and gender model, participants who reported being aged 65 years or older were 7.11 times more likely to belong to the very high and stable ([*OR* = 7.11; 95%*CI* = 1.38–36.57; *p* = 0.019) sleep quality trajectory group than participants reported being 18 to 25 years old. Additionally, participants who identified as male were 2.17 times more likely to belong to the high and stable (*OR* = 2.17; 95%*CI *= 1.15–4.10; *p* = .017) sleep quality trajectory group than participants who identified as female. In the model containing all other predictors, participants who reported following the government’s recommendations were 1.41 and 2.02 times more likely to belong to the high and stable (*OR* = 1.41; 95%*CI *= 1.01–1.96; *p* = .043) and very high and stable sleep quality trajectory groups (*OR* = 2.02; 95%*CI* = 1.28–3.17; *p* = .002), respectively, than participants who did not. In the ethnic background model, the predictor was not statistically significant.

### Joint trajectories analysis of sleep quality and sleep duration during the first wave of the COVID-19 pandemic

Table 6 shows the probabilities of belonging to each trajectory of one sleep outcome, conditional on belonging to each trajectory of the other sleep outcome. The joint trajectory analysis results (see Table 6) indicate that the likelihood of belonging to the long and decreasing sleep duration trajectory group for those who belonged to the very high and stable sleep quality trajectory group was statistically significant at 98.9% (*p* < .001). No other associations were significant.

**Table 6:** Joint trajectory analysis results

Group	Low and Stable Sleep Quality	Moderate and Stable Sleep Quality	High and Stable Sleep Quality	Very High and Stable Sleep Quality
Probability of Sleep Quality Group Conditional on Sleep Duration Group				
Short and Stable Sleep Duration	.220	.659	.118	.003
Long and Decreasing Sleep Duration	.009	.214	.619	.159
Probability of Sleep Duration Group Conditional on Sleep Quality Group				
Short and Stable Sleep Duration	.928	.612	.089	.011
Long and Decreasing Sleep Duration	.072	.388	.911	.989***

*Note.* ***p-value < .001

## Discussion

In our study, we sought to understand the unique impact of COVID-19 on health by examining the intraindividual variations in sleep quality and sleep duration over time during the first wave of the pandemic in the Canadian adult population and exploring which individual characteristics predict unstable sleep. A handful of Canadian studies previously reported a decrease in sleep quality and duration, but none of those studies measured sleep longitudinally throughout the first wave of the pandemic and were representative of the population. In contrast, our study uses a longitudinal design and a representative sample. Our results point to a very small decrease in sleep duration, which was limited to two-thirds of our sample, and no changes in sleep quality throughout the first wave of COVID- 9. Furthermore, younger age and living with someone predicted a greater likelihood of belonging to an unstable sleep duration trajectory.

Overall, our results align with findings from other Canadian studies rather than those of studies conducted abroad, pointing toward a general decrease in sleep duration rather than an increase, albeit also to a stable sleep quality instead of a decrease. The observed stability of sleep quality differed from the findings reported in Morin and colleagues’ study [19], which reported a sleep quality decrease during the first COVID-19 wave (April – May 2020) compared with 2017–2018 prepandemic data. However, their sample was subject to a selection bias, as it consisted mostly of residents from the province of Québec, where additional COVID-19 policies were implemented, such as travel restrictions and non-essential business closures [27, 28], likely increasing the burden of COVID-19 on daily life, and a high proportion of insomniacs, who are likely to report worse sleep. They reported a decrease in sleep quality during the first wave of the pandemic compared with prepandemic times. An increase in the emergence of sleep difficulties during the first COVID-19 wave (April 3 – June 24) in comparison with preoutbreak times (1 month before the outbreak) was measured retrospectively by Robillard et al. [25] This finding was also observed in other studies conducted worldwide using retrospective sleep measurement tools referring to different prepandemic times [13, 20, 73]. However, retrospective measures may overestimate the impact of the pandemic on sleep as they introduce recall bias. Alternatively, in our study, we observed exclusively stable sleep quality trajectory groups.

Instead of an overall increase in sleep duration during the first wave of the pandemic in comparison with prepandemic data as reported abroad [13, 16, 17, 74], we observed that sleep in Canada was mostly stable for one-third of our sample and decreased slightly for the other two-thirds. A study by Carroll and colleagues [26] conducted on Canadian families during the first COVID-19 wave also found that sleep was mostly stable in approximately half of their sample and decreased in the other third between the start of the COVID-19 pandemic (“since COVID-19”) and April–May 2020. However, their findings were limited by the retrospective nature of their prepandemic data and limited to the province of Ontario, which implemented additional non-essential business closure policies unseen in most provinces of Canada [28]. Robillard et al. [25] also reported a decrease in sleep duration during the first COVID-19 wave (April 3 – June 24) in comparison with preoutbreak times (1 month before the outbreak), although they did not report whether this decrease was led by a subgroup of participants or across their entire sample. Our results extend the findings of Robillard et al. and Carroll et al.’s works, as they provide a more complete understanding of the two different trajectories of sleep duration reported by the public during the first wave of the pandemic. This discrepancy between local and foreign studies regarding sleep duration changes could be due to the retrospective nature of prior studies, which might reflect erroneous location-specific participant beliefs about the effects of COVID-19 on their sleep. Furthermore, our results support that being older than 25 years predicts a stable sleep duration trajectory, which is in line with previous studies conducted worldwide during the first COVID-19 wave, which reported that old age is a protective factor for sleep health. [29, 32, 33] Nevertheless, contrary to what was hypothesized, living alone or with minors was not shown to predict an unstable sleep duration trajectory. The results from Morin and colleagues’ study [19] instead support that living alone was associated with increased fatigue and that lower social support was associated with more severe insomnia and poorer sleep during the first wave of the pandemic. This discrepancy between these findings may be explained by the generalizability of their results, as their data were obtained from a larger study designed to investigate a sample of individuals with insomnia, which may be subject to selection bias.

Our results suggest that sleep is overall stable in the context of the COVID-19 crisis, suggesting that sleep was more likely governed by factors that remained stable throughout the pandemic, such as environmental signals that entrain circadian rhythms (i.e., zeitgebers), such as light exposure [75], meal timing [76], and some aspects of social routines [77], such as online interactions and in-person household interactions, rather than other contextual environmental factors, such as being confined to one’s home. The remarkably small number of significant predictors of unstable sleep that we observed could reflect the stabilizing power of these factors. Subsequent research could investigate the underlying causes of the overall stability of sleep that was observed during the first wave of the pandemic. The heterogeneity between the two sleep duration trajectory groups (one stable, the other unstable) may be explained by the competing effects of home confinement or remotely performed activities. As suggested by Altena et al. (2020), [78] home confinement can likely be detrimental to sleep duration since it forces individuals to commit to various activities that might deprive them of sleep time, such as house administration, homeschooling, and household errands, and burdening them with additional stress. However, home confinement can also be beneficial to sleep, as time normally spent at work and in transit to work is likely reduced due to teleworking and business closures, freeing more time for sleeping but also for engaging in healthy behaviors beneficial to sleep and allowing more freedom to sleep according to one’s chronotype [78]. The addition of a measure of subjective freedom to sleep would allow a better understanding of this sleep duration disparity. The stability of sleep may also be linked to the characteristics of the population most at risk from the viral threat and the public health measures. During the pandemic, there was a notable decline in health services particularly for residents of long-term care facilities, who represented 67% of COVID-19-related deaths in Canada as of February 15, 2021 [79]. In contrast, individuals living outside long-term care facilities may not have faced the same disruptive changes to their quality of life, as they resided outside of these restrictive environments. Our study revealed several factors that were protective of sleep duration stability. The first is older age, which has been suggested to increase resilience to crises due to the slower pace of life and reduced social and economic pressure that accompanies an old age lifestyle, along with the use of past coping skills acquired from life experiences to address fear and uncertainty [80]. Some studies support that having a sense of being able to successfully adapt to challenging experiences (resilience) is a potential buffering factor against sleep disturbances and poor sleep quality [81, 82]. Belonging to the oldest age group (65 +) was also associated with reporting the highest sleep quality, further supporting the protective role of age on sleep. The second factor was living alone, which was not associated with a greater likelihood of an unstable sleep duration trajectory. Living with someone might reflect the presence of family responsibilities associated with the emergence of new sleep difficulties during the first wave of the pandemic. Family responsibilities might increase the likelihood of the occurrence of relational conflicts, which are reported to be rising worldwide during the pandemic [83], and bed-sharing, both of which are associated with poorer sleep outcomes [84–86]. Living alone might also decrease the risk of contracting COVID-19 through household transmission and alleviate pandemic-related life disruptions. Living alone might also not have impacted social life significantly during COVID-19, as social connectedness rose during the pandemic [87]. The addition of a measure of family responsibilities would shed light upon the mechanism driving the protective effect of living alone on sleep. In contrast, those in the long and unstable sleep duration group were more likely to belong to the highest sleep quality trajectory group. This might be attributable to the reported reduction in changes in daily routines between work days and free days due to the implementation of stay-at-home orders and the transition to telework during the first wave of the pandemic [88, 89]. Sleep tends to be insufficient during work days or constrained to a schedule that is maladapted to an individual’s chronotype, leading to an accumulation of sleep debt, but is usually recovered during free days, as sleep is allowed to last longer and schedules are looser, a phenomenon known as social jet lag [90]. Accordingly, COVID-19-related social restrictions might have reduced social jet lag by allowing more freedom to sleep according to one’s needs, thus allowing them to repay their sleep debt and perceive a higher quality of sleep. As sleep debt is repaid, sleep duration decreases over time. This is known as sleep satiation. Having access to prepandemic data for comparison with pandemic data would allow researchers to verify that hypothesis by revealing whether this decrease in sleep duration followed a sudden increase in sleep duration following the earliest pandemic disruptions. The results also highlight that ethnicity, overall, was not a predictor of deficient sleep. Our study has several strengths. Our study was the first to collect sleep data throughout the first wave of the pandemic on a biweekly basis throughout Canada, a critical time that was marked by the early days of the pandemic. Our study was also the first to recruit a representative sample from Canada through a quota-based sampling method. This sample reflected the adult Canadian population in terms of age, gender identity, province of residence, household size, current occupation, and country of origin [55]. Our analysis also reveals that our final sample was representative in terms of ethnicity (see Table 3). Additionally, identifiable sociological and demographic variations in our sample were also mitigated through a weighting process using census data from Statistics Canada [57, 64], based on the presence of household members under 18 years old, on the province of residence, and aboriginal background, improving the representativeness of our sample. Moreover, this study is also the first longitudinal study to survey sleep more than twice in a Canadian sample during the first wave of COVID-19. Finally, our study was the first to use latent class growth analysis to detect potential sleep duration and quality trajectories during the first wave of the pandemic throughout Canada. This methodology allowed us to determine subtle patterns of change in sleep outcomes over time and to distinguish subgroups following different patterns of change over time, when applicable.

Our study has several limitations. The use of sleep quality and sleep duration items based on the last 24 h may not have been representative of the last two weeks; most commonly validated sleep questionnaires tend to assess a period between one week and 4 weeks [91]. However, there were no differences between weekday and weekend responders, and Levene’s test indicated that there were no significant differences in the variances between weekday and weekend responders. These results suggest that the day of the week most likely did not significantly impact the survey results. Furthermore, our findings may not apply to all populations, as they excluded individuals without internet access and those who could not understand English or French. However, these populations are estimated to represent 6% [92] and 1.8% [93], respectively, of the Canadian population. Additionally, despite being representative of the Canadian population in terms of age, gender identity, province of residence, ethnicity, household size, current occupation, and country of origin, our final sample may not have been representative of other sociological and demographic variables. Of note, as reported previously [55], Canadians with lower levels of education (for instance, 45.7% without bachelor’s degree in sample, 71.5% in Census data), native French speakers (Sample 16.6%, Census 21,1%), and Indigenous people (Sample: 2.7%; Census 4.9%) were underrepresented in our original sample according to the Statistics Canada 2016 census profile. We attempted to mitigate this limitation by using a weighting process to correct identifiable sociological and demographic imbalances, utilizing data from 2016 from Statistics Canada [57]. However, use of quota-based non-probabilistic sampling may increase unmeasured heterogeneity into our sample, and thus potentially impact our results. The use of a weighting technique to address disparities between our sample and the Canadian population may not reduce selection bias for variables that were weakly associated with sociological and demographic factors, thus affecting the generalizability of our findings. As shown by Haddad et al., [94] the use of weighting to correct for sociological and demographic features of the sample may not necessarily change results. Taken together, it is therefore possible that differences between the original sample and the Canadian population may have persisted, decreasing the generalizability of our findings. Furthermore, since participants were required to be Canadian citizens or permanent residents, our study does not include temporary workers, who were estimated to represent approximately 2.9% of the population in 2017 [95]. However, this proportion might have been reduced during the first wave of the pandemic due to travel restrictions and a sudden slowdown in economic activity. Mental health problems that impact sleep, such as depression or Post-Traumatic Stress Disorder, were also not measured because the goal of our survey was to gain a better understanding if the psychological impact of COVID-19 in general and did not target sleep specifically. Finally, the findings in this study are possibly limited to crises similar in nature and scale to the COVID-19 pandemic, and may not extend to other crises, such as individual-level crises.

## Conclusion

This study is the first to provide empirical evidence on sleep trajectories and their health-related predictors during the first wave of the COVID- 9 pandemic utilizing a large representative sample of Canadians. Our findings that sleep duration and sleep quality remained stable overall throughout the first 4 months of the pandemic support the notion that sleep is governed by a constellation of factors that lead to stable and resilient sleep, even in the context of a crisis. Similarly, we found that people living with others were more likely to report longer sleep durations and a slight decrease in sleep duration over time. Furthermore, adults aged 25 years or older were less likely to belong to the unstable (longer sleep) trajectory. Future studies should examine whether similar results are observed in more diverse samples.

## Supplementary Information

Supplementary Material 1.

## Data Availability

In the method section, the data after filtering out participants who did not answer to both sleep outcomes at least 3 times each is available on https://osf.io/kcwd4/?view_only=be4ab33eba304a978272d512850daec8 along with the R, SPSS and SAS code used. The study design and analysis were not pre-registered.

## Abbreviations

BIC: Bayesian Information Criteria
FIML: Full Information Maximum Likelihood
MCAR: Missing Completely At Random
