# Supplementary material for: Sleep duration and quality trajectories during the early days of the COVID-19 pandemic: a Canadian nationally representative study
Source: BMC Public Health. 2025 May 7;25:1691. doi: 10.1186/s12889-025-22617-3 (PMC12057171; doi:10.1186/s12889-025-22617-3)
Supplement: Supplementary file 1 — Supplementary Material 1. [file 12889_2025_22617_MOESM1_ESM.docx]

**Sleep Duration and Quality Trajectories During the Early Days of the COVID-19 Pandemic: A Canadian Nationally Representative Study**

Anthony Levasseur^a,b^, Mathieu Pelletier-Dumas^c^, Éric Lacourse^d^, Jean-Marc Lina^e^, Guido Simonelli^a,b,f*^, Roxane de la Sablonnière^c *^

1. Université de Montréal, Department of Medicine, H3T 1J4, Canada
2. Centre intégré universitaire de santé et de services sociaux du Nord-de-l’Île-de-Montréal, Hospital of the Sacred-Heart of Montreal, H4J 1C5, Canada
3. Université de Montréal, Department of Psychology, H3T 1J4, Canada
4. Université de Montréal, Department of Sociology, H3T 1J4, Canada
5. École de Technologie Supérieure de Montréal, Department of Electrical Engineering, H3C 1K3, Canada
6. Université de Montréal, Department of Neuroscience, H3T 1J4, Canada

*Denotes joint senior authorship

**Corresponding author: Guido Simonelli, Hospital of the Sacred Heart of Montreal, 5400 Boul. Gouin Ouest, Montreal, H4J 1C5, QC, Canada.**

Anthony Levasseur: [anthony.levasseur@umontreal.ca](mailto:anthony.levasseur@umontreal.ca)

Mathieu Pelletier-Dumas: [m.pelletier-dumas@umontreal.ca](mailto:m.pelletier-dumas@umontreal.ca)

Éric Lacourse: [eric.lacourse@umontreal.ca](mailto:eric.lacourse@umontreal.ca)

Jean-Marc Lina: [jean-marc.lina@etsmtl.ca](mailto:jean-marc.lina@etsmtl.ca)

Guido Simonelli: [guido.simonelli@umontreal.ca](mailto:guido.simonelli@umontreal.ca)

Roxane de la Sablonnière: [roxane.de.la.sablonniere@umontreal.ca](mailto:roxane.de.la.sablonniere@umontreal.ca)

# Abstract

**Background**

Poor sleep health has wide-ranging consequences for general health. The year 2020 marked the first year of the COVID-19 pandemic throughout the world, an event that introduced dramatic disruptions to daily life. Studies conducted during the first wave of the pandemic reported a decrease in sleep quality but also an increase in sleep duration, which contradicts the simultaneous decrease in sleep duration reported in Canada. However, prior studies were not representative of the Canadian population. To assess pandemic-induced health disruptions, we investigated sleep health trajectories and health correlates during the first wave of COVID-19 in a longitudinal nationally representative sample of Canadians. We aimed (1) to determine the trajectories of sleep duration and sleep quality, (2) to identify health factors associated with unstable sleep trajectories, and (3) to explore associations between sleep trajectory groups.

**Methods**

A nationally representative sample of 2,246 individuals residing in Canada was surveyed 6 times between April and July 2020. Participants reported on their sleep and health-related factors (e.g., sociological and demographic factors). We first used latent class growth analysis to identify sleep trajectories. We then used multinomial logistic regression models to determine the relationships between health-related predictors and trajectory groups. Finally, we used joint trajectory analysis to explore the relationships between sleep duration trajectories and sleep quality trajectories.

**Results**

We identified four constant sleep quality trajectories (6.7%, 37.1, 45.5%, and 10.7% of the sample). We identified two sleep duration trajectories, one of stable short sleep (33.9%), and one of decreasing (-2.32 min/2 weeks) long sleep (66.1%). Living with someone predicted longer and decreasing sleep duration. Being 25 or older was associated with a lower likelihood of belonging to the longer and decreasing sleep duration trajectory. There was a 98.9% likelihood of belonging to the longer and decreasing sleep duration trajectory for those belonging to the higher sleep quality trajectory.

**Conclusions**

In our study, we found no convincing evidence that sleep health indicators deteriorated during the first wave of COVID-19 in Canada. The overall stability of sleep suggests that sleep is likely governed by factors that remained stable.

**Keywords: Sleep, Longitudinal, Trajectory, COVID-19, Health, Predictor, Correlates, Canada, Mental Health, Environment**

# Abbreviations

BIC: Bayesian Information Criteria

FIML: Full Information Maximum Likelihood

MCAR: Missing completely at random

# Background

Poor sleep health has far-reaching and wide-ranging consequences for physical and mental health. According to a 2022 umbrella review of 85 meta-analyses, highly suggestive evidence supports the association between long sleep duration and an increased risk of all-cause mortality, and suggestive evidence supports the association between short sleep duration and an increased risk of overweight and/or obesity, as well as poor sleep quality and an increased risk of diabetes mellitus ^1^. Poor sleep quality has also been causally related to mental health difficulties. ^2^ Insomnia has been identified as a predictor of suicidal thoughts and behaviors, ^3,4^ anxiety, ^5,6^ and later onset of psychopathologies such as depression and psychosis. ^7,8^ Short (<7h/night) and long (>9h/night) sleep durations are also associated with a higher risk of cognitive disorders. ^9^ Disrupted sleep negatively impacts mental health in adults, ^10^ and poor sleep quality is causally related to the experience of mental health difficulties. ^2^ In Canada, short sleep and poor sleep quality are highly prevalent, with approximately one-quarter of Canadian adults reporting not receiving sufficient sleep and up to one-fifth not finding their sleep “refreshing”. ^11^

The year 2020 marked the first year of the COVID-19 pandemic, an event that introduced dramatic social changes ^12^ into daily life throughout the world. Many studies have reported an increase in the prevalence of sleep problems around the globe during the COVID-19 pandemic between the prepandemic period, generally referred to as before January 30^th^ 2020, and June 2020. These studies reported a decrease in sleep quality, ^13–15^ an increase in sleep duration, ^14,16,17^ and an increase in insomnia symptoms. ^18,19^ However, there was no consensus regarding what period corresponds to “prepandemic” sleep, as many studies invited participants to set their own reference for prepandemic sleep which may not correspond to the same period throughout their sample. This may encourage participants to compare their current sleep with the period they associate with having the best sleep, even if it was impacted by the pandemic in some way, thus failing to measure the pandemic’s effects on sleep. The aforementioned studies also used retrospective measures of prepandemic sleep, exposing themselves to recall bias. Participants might be inclined to perceive their prepandemic or early-pandemic sleep favorably, as they might believe that their sleep was impacted during the pandemic by the measures in place. A longitudinal study by French et al. ^20^ measuring sleep quality both in late March 2020 and a month later also reported a decrease in sleep quality between these time points. However, it relied on a retrospective measure of sleep quality ("in the last 3 months"), which is susceptible to recall bias. It is suggested that these changes were at least partially driven by the widespread COVID-19-related disruptions of daily life, such as home confinement, changes in work and social habits, and threats to health caused by the virus. ^21,22^ In Canada, during the first wave of the pandemic (broadly defined as the period between late January to the end of June or mid-July of 2020 ^23,24^), a handful of studies reported on sleep health outcomes, reflecting a sleep quality decrease as well, but contradictory results regarding changes in sleep duration. A retrospective increase in the emergence of sleep difficulties and an average decrease in sleep duration was reported by Robillard et al. during the first COVID-19 wave (i.e. in the 7 days before filling out the survey) in comparison with preoutbreak times (i.e. in the last month before the outbreak). ^25^ A retrospective decrease in sleep duration between the start of the COVID-19 pandemic (“since COVID-19”) and April–May 2020 was also reported by Carroll et al. in 34% of their adult participants. ^26^ Morin et al. also reported a decrease in sleep quality and an increase in insomnia between 2017-2018 and April–May 2020. ^19^ However, none of these studies were representative of the Canadian population, as Robillard et al.’s study included a large proportion of white, highly educated, high-income, highly employed, middle-aged women; Carroll et al.’s study was limited to parents of families from the province of Ontario; and Morin et al.’s study was limited to French-speaking Canadians, residing mostly in the province of Quebec. The samples in Robillard et al.'s and Carroll et al.'s studies may be characterized by greater resource availability, which could enable better coping with the negative impacts of crises. This may mitigate mental health and sleep health issues, potentially limiting the generalizability of their findings. Morin et al’s sample was predominantly from Quebec, a region in which additional COVID-19 policies were implemented, in comparison to the rest of Canada, such as closures of non-essential businesses and regional travel restrictions, ^27,28^ likely increasing the burden of the pandemic on daily life. To evaluate the impact of the COVID-19 pandemic on Canada's population, it is essential to gather a sample that reflects its demographics in terms of age, gender, and province of residence, thereby enhancing the representativeness of the findings. To achieve this, our study will choose participants according to established quotas for these three sociological and demographic variables and apply weighting adjustments to address any identifiable sociological and demographic discrepancies in our sample. The use of different questionnaire items may also explain the discrepancies between prior Canadian and foreign studies, as nearly half of the aforementioned studies used researcher-developed tools or device-based measures.

During the early days of the pandemic, several studies attempted to identify factors associated with sleep disruption. Studies conducted worldwide have revealed associations between poorer sleep health and various sociological, demographic, environmental, biological, and subjective exposures to COVID-19 factors, such as younger age, ^20,29–34^ identifying as a woman, ^30,34–39^ low social support, ^19^ living with children, ^25,39–41^ being isolated or quarantined, ^42,19,33,38,43^ being exposed to a high COVID-19 threat, ^40,44^ working in healthcare, ^39,40,45^ being concerned with COVID-19, ^30,33,34,46^ being diagnosed with COVID-19, ^40,43,47^ and experiencing financial difficulties. ^19,33,39,40^ Being a racialized minority (e.g. being a member of a Black or Asian ethnic group) in the United States of America and in the United Kingdom was also associated with being more vulnerable to sleep health disparities during the first wave of the COVID-19 pandemic. ^39,48^ In Canada, visible ethnic minority groups are defined as “'persons, other than Aboriginal peoples, who are non-Caucasian in race or non-white in color”. ^49^ However, it was reported that Aboriginal peoples were eight times more likely to die from COVID-19 compared to non-aboriginal individuals in Canada, ^50^ with high levels of overcrowding, inadequate housing, and limited access to healthcare services documented as significant factors contributing to the increased risk of transmission and severe illness. ^51^ This suggests that belonging to an ethnic minority group, or to an Aboriginal people, may predict worse sleep in Canada.

However, an important limitation of this body of work is that most of these studies had cross-sectional designs, used retrospective data, and used sleep items that referred to different periods as “prepandemic”. Accordingly, cross-sectional sleep data may not capture lifestyle changes impacting individuals, particularly in the context of a rapidly evolving environment and fast-changing policies that may include income support, border control, travel, transport, and quarantine measures. ^52^ In contrast, a longitudinal assessment of sleep during the early days of the pandemic could identify changes in sleep (i.e. unstable sleep), that may have been pandemic-induced disruption. To date, no published study has surveyed sleep duration and quality multiple times during the early days of the COVID-19 pandemic and explored predictors of sleep health outcomes in a representative sample of Canadians.

Sleep duration and sleep quality are key concepts of sleep health. ^53^ Sleep duration and quality are both distinct in that they can impact health differently but are also inextricably linked, as individuals with short and long sleep durations are also those most likely to report sleep disturbances, ^54^ which is indicative of poor sleep quality. The evidence suggests that the effects of sleep duration and sleep quality on health outcomes are also not simply additive, calling for measurements of both aspects of sleep when possible. ^54^ This evidence highlights the need to determine the interplay, if any, between sleep duration and sleep quality.

To address the limitations outlined above, we present the first longitudinal investigation of sleep trajectories and their relationships with COVID-19-related factors in a representative sample of Canadians during the first wave of the COVID-19 pandemic. The present study aims to (a) determine sleep quality and sleep duration trajectories in our sample during the first COVID-19 wave; (b) identify COVID-19-related sociological, demographic, environmental, biological, and other exposure predictors of unstable sleep quality and duration trajectories; and (c) determine associations between sleep quality and sleep duration trajectories. Determining sleep trajectories during the first wave of COVID-19 would allow for a better understanding of the immediate impact of a public health crisis of this scale on sleep and an assessment of its evolution. A better understanding of the predictors of sleep health trajectories and associated sleep trajectories could be used to develop better sleep programs to mitigate the impact of public health crises comparable to COVID-19 on sleep health. For instance, another viral outbreak of the same scale as COVID-19 in the future could lead to home confinement measures once again. Knowing which populations are most vulnerable to the effects of home confinement on sleep health in advance may encourage policy adjustments for these populations. We hypothesized that (1) sleep quality and sleep duration were both unstable in at least one trajectory group per outcome variable and that (2) younger age, identified as a woman, living alone or with minors, reporting a higher local COVID-19 spread speed, staying at home frequently, being in voluntary isolation, being employed in healthcare, being very concerned about getting very sick with the virus, being concerned about peers getting very sick with the virus, having been diagnosed previously with the virus, having peers that have been diagnosed previously with the virus, being concerned about the financial impact of COVID-19 on oneself, reporting any impact of the crisis upon one’s personal life, following the government’s COVID-19 recommendations, identifying to a visible minority group of Canada (African-American, Latino or Hispanic, Asian, or other than White/Caucasian) or to an Aboriginal People significantly predicts unstable sleep quality and sleep duration trajectory group membership, and that (3) belonging to an unstable sleep duration trajectory group predicted belonging to an unstable sleep quality trajectory and vice-versa.

# Participants and Methods

## Study Setting

This study is part of a larger ongoing study that seeks to comprehend the overall impacts of the COVID-19 pandemic on the Canadian population. ^55^ The goals of the COVID-19 Canada Survey are to be better prepared for a potential subsequent public health crisis and to maximize the overall resilience of our society. This study makes use of 6 measurements spread over 14 weeks. This study provides an interim analysis of sleep duration and quality data collected during the first measurement times. The IRB approval for this study was given by the Research Ethics Committee in Education and Psychology of the University of Montreal. ^55^

## Data Collection

This longitudinal nonparametric study on Canadians was conducted during the first wave of the pandemic (see Figure 1)^23,24^. To capture the effects of COVID-19 on sleep during the first wave, we aimed to collect data during the peak of infection. To that end, we collected data between April 6^th^ and July 13^th^ of 2020 (see Figure 1). Data was collected through the *AskingCanadians* (French: *Qu’en pensez-vous*) web panel from the survey firm Delvinia ^56^ which recruited Canadian adults. The firm used SMS messaging and mobile app reminders to reduce attrition and was carried out online in French and English. Delvinia selected participants based on established quotas from the 2016 Census Profile ^57^ of Statistics Canada for three sociological and demographic variables: age, gender identity, and province of residence (for details, see COVID-19 Canada’s Technical Report ^55^). In the survey, eligible participants were invited to complete a questionnaire measuring sleep health predictors once during the first measurement time and to complete a questionnaire measuring sleep outcomes 5 subsequent times (see Figure 2). Each survey given to participants included a welcome paragraph along with a consent form. Informed consent was given by all participants prior to completing each survey. Participants received a compensation of approximately 2.50 Canadian dollars per completed questionnaire, with the payment provided as points that could be redeemed at partner companies of their choice. The survey used a rolling cross-sectional survey design. ^58^ Every day during each measurement time (14 days total per measurement time), a subsample of participants drawn from the first (largest) measurement time sample was contacted to complete the survey until a daily target of approximately 250 participants was reached. When contacted, participants had 7 or 14 days to complete the survey (see Table 1). The 14 subsamples were always contacted 14 days apart from their last invitation. The completion time per questionnaire ranged from 15 to 20 minutes. Participants who missed one or more waves were able to participate again at any subsequent measurement time. A procedure of planned missingness was implemented across three of our predictor variables but not our outcome (sleep) variables. ^59,60^ The three predictors were “Concern for self getting very sick with the virus”, “Concern for a peer getting very sick with the virus”, and “Concern about the financial impact of COVID-19 on self”. For these predictors, only 66% of the participants were able to provide answers. According to Enders, ^61^ the utilization of planned missingness allows for the maintenance of validity and statistical power without any compromise. This procedure consists of the presentation of questionnaire items only to subsets of participants to reduce the length and cost of the data collection process. ^59^ Therefore, the questionnaire items were not always presented to the same participant from one measurement time to the other, leading to a large increase in missing data. The predictors of sleep health were measured only once at measurement time 1, before our outcome variables. The sleep variables were measured 5 times, at measurement times 2 to 6. To mitigate the potential impact of question position contamination, the questions were organized into 10 blocks, each addressing specific COVID-19 issues, and were administered in a randomized order. ^55^

1. *Weekly COVID-19 Cases and Death Count in Canada*


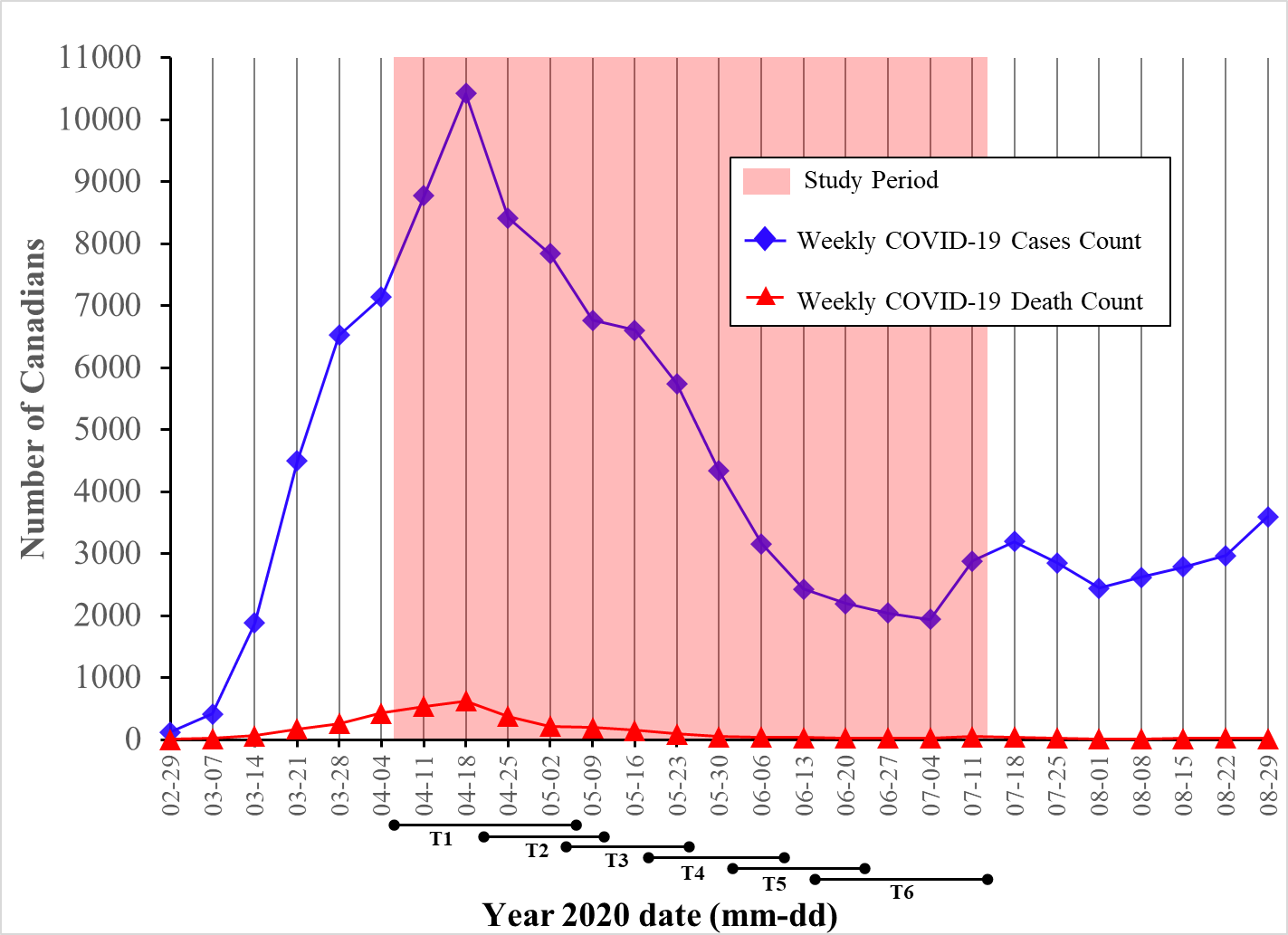


*Note*. Figure depicting the evolution of weekly COVID-19 cases and death count in Canada during the first measurement time of the COVID-19 pandemic, with the study period highlighted in red, and study assessment periods underlined under the graph. T1 – T6: Study measurement time 1 to 6. Figure built based on data collected from the *Public Health Agency of Canada* ^62^.

1. *Survey Timeline*


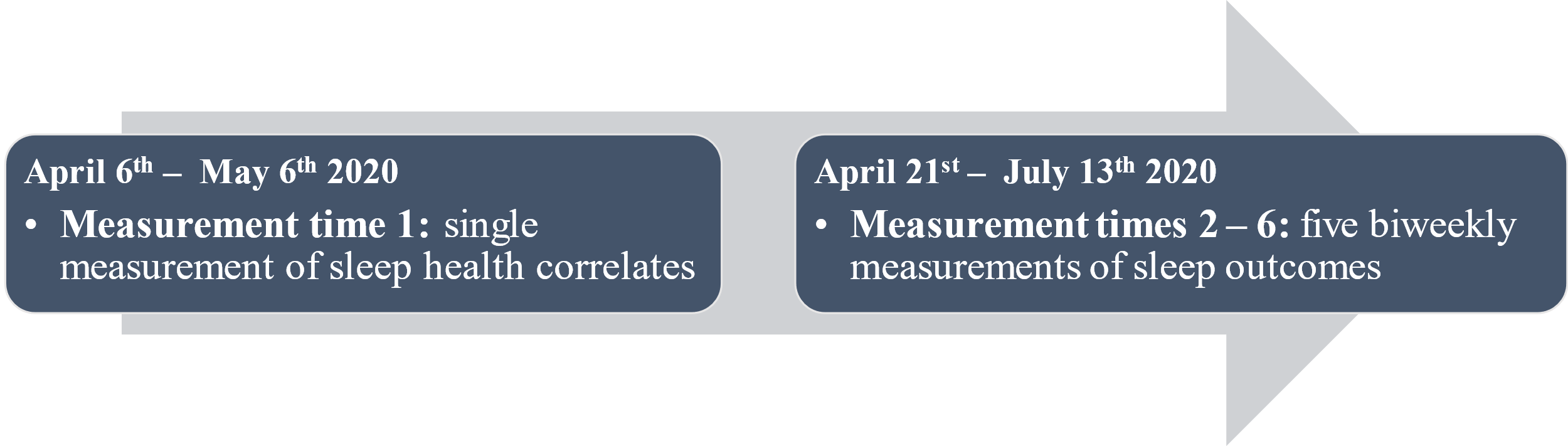


*Note*. Illustration of the series of measurements performed to gather the dataset utilized in this study, along with the corresponding time duration for each measurement time point.

## Participants

### Inclusion and Exclusion Criteria:

To be eligible, participants were required to be at least 18 years old and to be Canadian citizens or permanent residents. A total of 3,617 Canadians were recruited at the first measurement time of the survey.

To be included in our analysis, participants had to have answered both sleep duration and sleep quality questions at least three times (minimum number of measurement times required to model a linear trajectory of change). Responses provided in less than 4 minutes and those associated with the failure of the two attention check items included within each survey during measurement times 2 to 6 were considered invalid, and they were excluded from the sample. Of the total number of responses, 3.7% and 0.2% were excluded due to the failure of both attention check items and the completion of a questionnaire in 4 minutes or less, respectively. Furthermore, we excluded 1,371 participants (37.9%) who did not answer questions at least three times regarding their sleep duration and/or quality, leaving 2,246 participants as the final sample.

### Representativeness of the Sample

On the first measurement time, the sample reflected the adult Canadian population as described by Statistics Canada’s 2016 census profile data ^57^ in terms of age, gender identity, and province of residence. The sample was also comparable based on variables that were not included in the quotas such as ethnicity (as shown by Table 3), as well as household size, current employment status, and country of origin. ^55^ While the full information maximum likelihood is utilized in our trajectory analysis, missing data caused by attrition can have a significant effect on both the results and the representativeness of the sample. Accordingly, to reduce the differences between our sample and the population of Canada, we employed a raking weighting method ^63^ to correct for identifiable sociological and demographic variations in our sample. To identify sociological and demographic variations, we utilized data from 2016 and 2020 from Statistics Canada. ^57,64^ Table 3 shows that our sample also reflected the population of Canada in terms of ethnic background, as none of the Phi coefficients exceed a value of 0.19, indicating negligible associations between the presence of the ethnic background and the data source (0 = study sample and 1 = Census data). We performed weighting based on the following benchmark variables: 1) presence of household members under 18 years old, 2) province of residence, and 3) Aboriginal background (Canadian Indigenous community members). The weighting procedure was conducted under the function “calibration” from the *Icarus* package in R software. The weighting process had a maximum weight range of 9.56, which enabled the creation of weights ranging from 0.5 to 10.06, with a mean weight of 1. The weighting process led to an 11.30% reduction in bias, according to the selected benchmark variables.

Table 1

Study Population Characteristics

| Measurement Time | Missing Data (%) | Sample Size (*N*) | % (n) Women | Mean Age (range) | Survey Date^a^ (year 2020) | No. of Days to Complete the Survey |
| --- | --- | --- | --- | --- | --- | --- |
| 1 | 0 | 2246 | 49.5 (1111) | 49.85 (68) | April 6 – May 6 | 14 |
| 2 | 16.4 | 1878 | 41.0 (921) | 50.39 (68) | April 21 – May 11 | 7 |
| 3 | 12.2 | 1974 | 43.4 (974) | 49.93 (68) | May 4 – May 25 | 7 |
| 4 | 11.6 | 1987 | 42.9 (963) | 49.84 (68) | May 18 – June 10 | 7 |
| 5 | 15.9 | 1891 | 40.8 (916) | 50.28 (68) | June 1 – June 23 | 7 |
| 6 | 16.5 | 1878 | 41.5 (933) | 50.30 (68) | June 15 – July 13 | 14 |

*Note*. ^a^ includes the number of days to complete the survey.

Table 2

*Sample Distribution According to the Territory of Residence*

| Measurement time | Maritimes^a^ | | British Columbia | | Ontario | | Quebec | | Prairies^b^ | | Newfoundland and Labrador | |
| --- | --- | --- | --- | --- | --- | --- | --- | --- | --- | --- | --- | --- |
|  | n | % | n | % | n | % | n | % | n | % | n | % |
| 1 | 135 | 6.0 | 320 | 14.2 | 919 | 40.9 | 439 | 19.5 | 403 | 17.9 | 30 | 1.3 |
| 2 | 122 | 5.4 | 276 | 12.3 | 777 | 34.6 | 335 | 14.9 | 340 | 15.1 | 28 | 1.2 |
| 3 | 111 | 4.9 | 286 | 12.7 | 809 | 36.0 | 395 | 17.6 | 347 | 15.4 | 26 | 1.2 |
| 4 | 119 | 5.3 | 296 | 13.2 | 811 | 36.1 | 385 | 17.1 | 350 | 15.6 | 26 | 1.2 |
| 5 | 124 | 5.5 | 274 | 12.2 | 793 | 35.3 | 321 | 14.3 | 355 | 15.8 | 24 | 1.1 |
| 6 | 117 | 5.2 | 259 | 11.5 | 786 | 35.0 | 358 | 15.9 | 332 | 14.8 | 26 | 1.2 |

*Note*. ^a^ New Brunswick, Nova Scotia, and Prince Edward Island. ^b^ Alberta, Manitoba, and Saskatchewan.

Table 3

*Sample and Census Ethnic Background Distribution*

| Ethnic Background | Sample data  (N=2,246) | | Canadian Census Data (2016) ^57^ | Pearson Chi-Square Goodness of Fit |  | Phi Coefficient^a^ |
| --- | --- | --- | --- | --- | --- | --- |
|  | n | % | % | χ² (1) | *p* | φ |
| White/Caucasian | 1393 | 62.0 | 72.9 | 11.93 | 0.001 | 0.08 |
| African-American | 23 | 1.0 | 3.5 | 23.65 | 0.000 | -0.07 |
| Latino or Hispanic | 22 | 1.0 | 1.3 | 0.02 | 0.892 | 0.00 |
| Asian | 237 | 10.6 | 15.0 | 2.26 | 0.133 | -0.02 |
| Aboriginal | 23 | 1.0 | 4.9 | 45.56 | 0.000 | -0.10 |
| Other/Unknown | 61 | 2.7 | 2.4 | 8.81 | 0.003 | 0.03 |
| Prefer not to say | 31 | 1.4 | NA | NA | NA | NA |
| Missing | 504 | 22.4 | NA | NA | NA | NA |

*Note*. ^a^ Association between the presence of the ethnic background and the data source (0 = study sample and 1 = Census data)

## Measures

### Sleep Outcomes

Our outcome variables, sleep duration and sleep quality, were measured 5 times from April 21^st^ to July 13^th,^ 2020. The participants were given a “Prefer not to answer” option every time.
According to the National Sleep Foundation, ^65^ to capture sleep quality, participants can rate their sleep as good, bad, or something in between. Thus, we assessed sleep quality with the question: “*How would you describe the quality of your sleep during the last 24 hours?*”. To capture a wide spectrum of sleep quality ratings, the participants answered a Likert scale ranging from 1 (“*slept very badly*”) to 10 (“*slept very well*”). Participants had to answer recall only the last 24 hours to mitigate recall bias.

The sleep duration item used similar phrasing to the validated Pittsburgh Sleep Quality Index. ^66^ Whereas the PSQI measures sleep duration with the question "*During the past month, how many hours of actual sleep did you get at night*", our item assessed sleep duration with the question: “*How much sleep did you get in the last 24 hours? (please insert the possibility for hours and minutes)*”. Participants entered the number of hours and minutes they had been asleep during the last 24 hours. Sleep duration was then converted to minutes.

### COVID-19-Related Predictors of Sleep Health

Our predictors consisted of sociological, demographic, environmental, biological, and other exposure factors to the virus. Table S1 in Additional File A1 details all predictors and their origins.

The “healthcare-related employment” predictor was determined based on the answer provided to the question, “*What is your current job or profession?*”. The responses were evaluated and categorized using Canada's National Occupational Classification, which is the standardized system for describing occupations in the country. ^67^ Subsequently, dummy coding was utilized to indicate whether the provided answer corresponded to a “health occupations” category.

## Statistical Analyses

All analyses were conducted using SAS version 9.4 software and the PROC TRAJ procedure. ^68,69^ Statistical significance was set at a bilateral alpha level of 0.05*.* We utilized Nagin's ^70^ semiparametric group-based modeling approach to fulfill the first objective and performed a latent class growth analysis with an intraclass variance fixed to zero. The growth model was based on a censored-normal distribution, ^70^ and time was coded in weeks, from 0 to 8. More specifically, the first measurement of sleep, which spanned 2 weeks (April 21^st^ – May 3^rd^), with an additional week for late responses (meaning that it could be answered on May 11^th^ at the latest for those meant to answer on May 3^rd^), was coded as 0. The second measurement of sleep, which started two weeks after the intended response time for measurement time 1, was coded as 2, and so on in increments of 2, until the final measurement time, coded as 8. First, we determined the optimal number of trajectory groups from one to five third-order trajectory groups by applying the requirement of a minimum of at least 5% of the sample (*n*=112) per group. ^71^ Second, we determined the optimal order for each trajectory group based on the Bayesian Information Criterion (BIC), keeping the model that had a value closest to zero. ^70,72^ Given the impossibility of specifying to the PROC TRAJ SAS package the real limits (between 1 and 10) of our distribution of sleep quality, all estimated trajectory parameters exceeding the maximum of 10 were adjusted to 10, and all values inferior to 1 were adjusted to 1. We defined ‘unstable sleep trajectory’ as a trajectory identified by latent class growth analysis with at least one statistically significant coefficient of a greater order than 0.

We performed a Pearson correlation matrix of all predictors except age and gender to reject multicollinear predictors that met or exceeded an absolute correlation coefficient value of 0.3 with each other. We performed multinomial logistic regressions using the “RISK” function to estimate the likelihood of being assigned to specific trajectory groups in comparison to the first trajectory group (i.e., the lowest sleep quality group or shortest sleep duration group) as a baseline based on individual-level factors (i.e., our COVID-19-related sleep health predictors). We computed 3 models per outcome variable: the first model utilized age groups and gender identity only as predictors, using the youngest age category (18-25 years old) as the baseline category, the second model utilized all other valid predictors except ethnic categories, and the third model utilized only “Non-White Ethnicity” as predictor, a binary variable representing participants that did not report “White/Caucasian” as one of their ethnic backgrounds. Participants that selected “Prefer not to answer” to answer the ethnicity item were treated as missing data and were not included in the “Non-White Ethnicity” category. This variable was created to account for the small number of cases of Non-White ethnic backgrounds (except Asian) and to avoid the use of multicollinear predictors. To capture nonlinear relationships between the predictor and the log odds of the dependent variables, “Age” was categorized into age groups of 10-year increments. The variables related to household size were dummy coded; “Plural household size” described participants who lived with someone, whereas “Presence of household members below [age]” described participants who lived with minors under 18 or 6.

Finally, we used the joint trajectory analysis approach created by Jones et al. ^68^ to build two models to test the associations between the trajectory groups of our two outcome variables.

### Missing Data Strategy

Missing data were assumed and treated as missing completely at random (MCAR) for predictors with planned missingness. Missing data was handled with the Full Information Maximum Likelihood (FIML) method, allowing us to include participants with missing data without replacing missing values on the variables used to create the trajectory groups. ^59,68,70^ FIML is currently considered a best practice method for dealing with missing data, regardless of the missingness mechanism. ^61^ However, no estimation was performed for missing data for other predictor variables because they either represented very small proportions of our sample [see Additional File A1 Tables S6-9] or did not meet all validity criteria for analysis. Furthermore, we treated “healthcare-related employment” predictor responses as missing data for answers that could not be categorized via Canada’s National Occupation Classification system. “Prefer not to answer” responses were also treated as missing data.

# Results

## Sample Characteristics

At measurement time 1, participants in the final sample were aged between 18 and 86 years old (M = 49.85; SD = 16.76), and the female gender identity represented 49.5% of participants (n = 1111; with 0% “other”) [see Additional File A1 Tables S6 and S8]. The distribution of participants by province of residence is displayed in Table 2. The ethnic distribution of our sample, along with Statistics Canada’s 2016 Census data, is displayed in Table 3.

## Sleep Behavior During the First Wave of the COVID-19 Pandemic

The mean sleep duration within 24 hours was 442.76 minutes (standard deviation [SD] = 75.70), while the mean sleep quality score was 6.87 (SD = 1.89). The distribution of sleep quality was negatively skewed, with less than 22% of participants reporting a sleep quality of 5 or less [see Additional File A1 figure S4], reflecting Statistics Canada’s reported 1 in 5 adults not finding their sleep refreshing. All values of sleep duration were plausible, although they exceeded Statistics Canada’s reported value of 1 in 3–4 adults with insufficient (7-9 hours/night) sleep, ^11^ with 58.4% of adults getting less than 7 hours or more than 9 hours of sleep on average. No outliers were detected. Sleep duration and sleep quality were significantly associated, with higher sleep quality being associated with longer sleep duration (r=.464, p < .001). Additional descriptive characteristics of the outcome variables are available in Additional File A1.

## Trajectories of Sleep Duration and Sleep Quality During the First Wave of the COVID-19 Pandemic

We identified four trajectory groups of sleep quality and 0 as the optimal order for each trajectory, meaning that the 4 trajectory groups were best described by constants. The chosen model (see Table 4 and Figure 3) shows that sleep quality remained constant and that participants reported, on average, a quality of 2.48 (6.7% of the sample), 5.44 (37.1% of the sample), 7.83 (45.5% of the sample) and 10 (10.7% of the sample) out of 10.

We identified 2 trajectory groups of sleep duration and optimal orders of 0 for the shortest duration trajectory and 1 for the longest duration trajectory. The chosen model (see Table 5 and Figure 4) suggests that 33.9% of the participants had a shorter sleep duration that averaged 369.18 minutes and remained constant, whereas the other 66.1% had a longer sleep duration of 486.53 minutes, which initially decreased linearly at a rate of 2.32 minutes per week.

Additional File A1 Table S11 displays the Bayesian Information Criterion (BIC) values and group sizes for model selection based on the number of trajectory groups and the order of the trajectories of the model with the optimal number of trajectory groups. Figures S1 and S2 in the Additional File show the individual trajectory models for all participants for each trajectory group identified, labeled by trajectory number. For increased readability, the colors selected are the same.

Table 4

*Coefficient Estimates for the Group-Based Trajectory Model of Sleep Quality*

| Trajectory Group | Estimate | SE |
| --- | --- | --- |
| 1. Low and stable | **2.48*** (intercept)** | 0.60 |
| 2. Moderate and stable | **5.44*** (intercept)** | 0.27 |
| 3. High and stable | **7.83*** (intercept)** | 0.15 |
| 4. Very high and stable | **10*** (intercept)** | 0.22 |

*Note.* *** p-value < 1e-3; SE, Standard Error.

1. *Selected Sleep Quality Model*

**
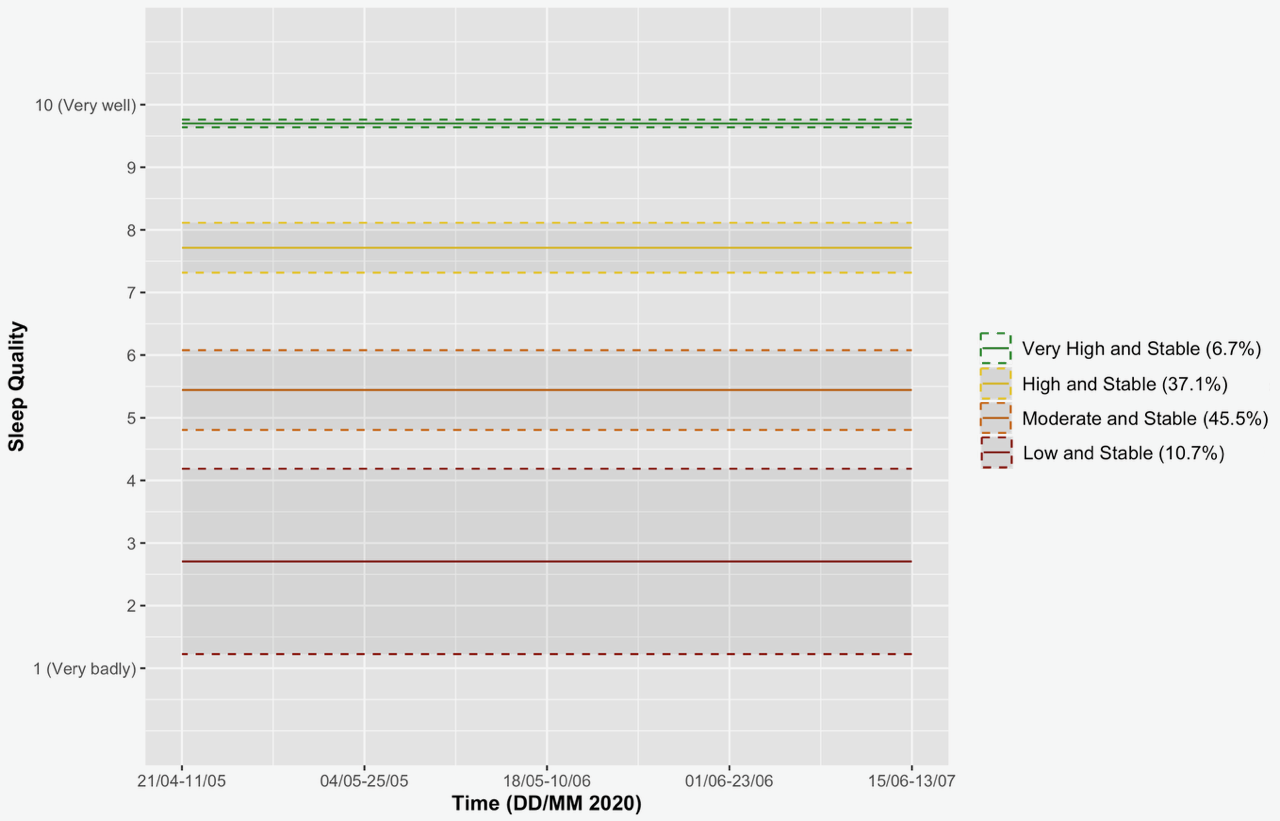
**

*Note*. Figure illustrating the selected sleep quality trajectory model across the 5 measurement times.

Table 5
*Coefficient Estimates for the Group-Based Trajectory Model of Sleep Duration*

| Trajectory Group | Parameters | Estimate | SE |
| --- | --- | --- | --- |
| 1. Short and stable | Intercept | **369.18*** (intercept)** | 12.73 |
| 2. Long and decreasing | Intercept | **486.53*** (intercept)** | 8.16 |
|  | Linear | **-2.32***** | 0.52 |

*Note.* *** p-value < 1e-3; SE, Standard Error.

1. *Selected Sleep Duration Model*


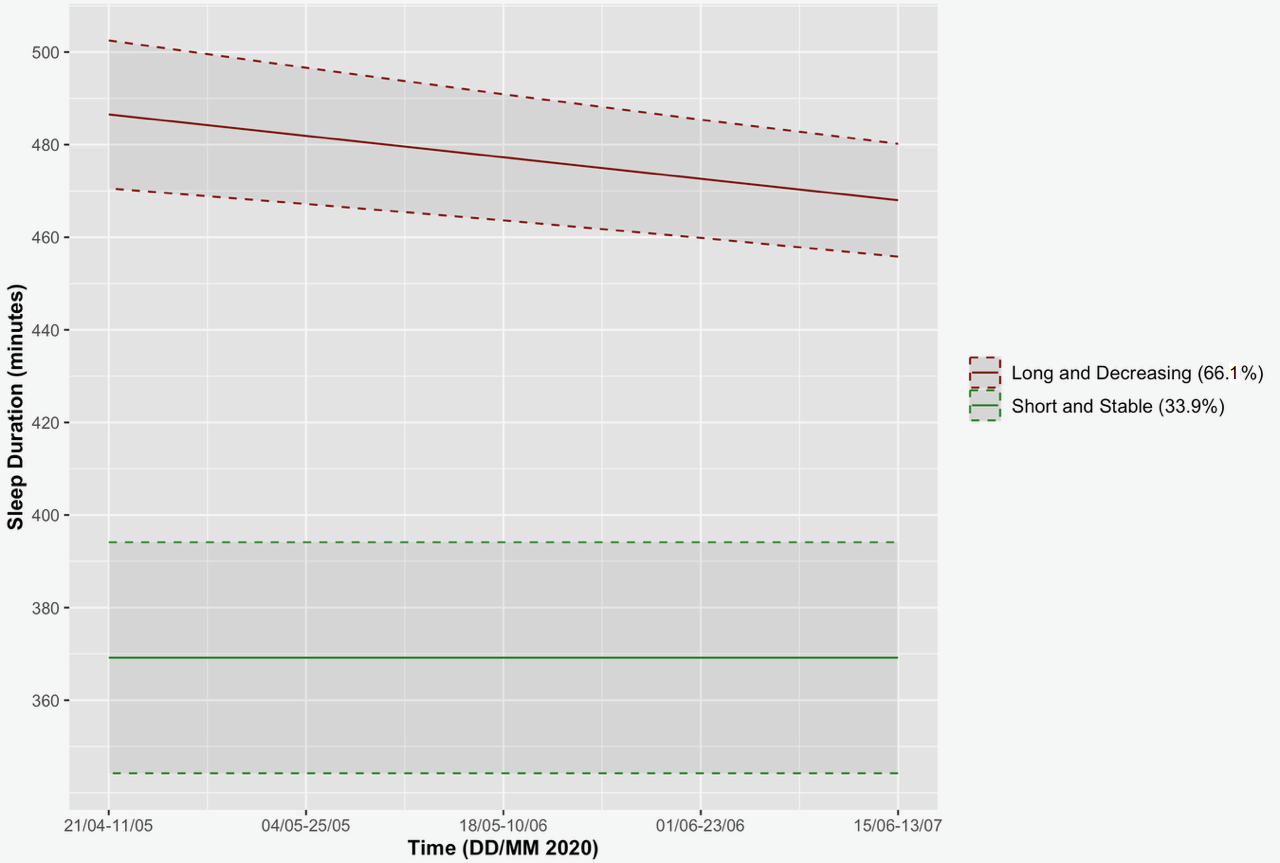


*Note*. Figure illustrating the selected sleep duration trajectory model across the 5 measurement times.

## Associations Between Sleep Trajectory Belonging and COVID-19-related Predictors of Sleep Health During the First Wave of the COVID-19 Pandemic

The predictors “healthcare-related employment” and “Self has been diagnosed with the virus previously” were removed from further analyses, as less than 5% of the participants answered each question positively (3.1% and 0.2%, respectively), thus leading to a lack of statistical power to compute the logistic regression model.

The Pearson correlation matrix (see Additional File A1 Table S10) identified five correlation values exceeding an absolute value of 0.3, leading to the rejection of “Concern for a peer getting very sick with the virus”, which was positively correlated (r = .615, p < .01) with “Concern for self getting very sick with the virus”, and “frequency of stay at home”, which was positively correlated (r = .451, p < .01) with “Self is following the government’s recommendations”. No action was taken for the correlation between Presence of Household Members Under 18 and Age group 35 to 45, as it was considered to be acceptable in magnitude (r = .304, p < .01). No action was taken for the correlations between ethnic category predictors as well, as they were replaced with the “Non-white” predictor. This intervention supports that the absence of statistical significance in individual predictors cannot be attributed to overlapping predictors.

To explore the relationship between predictors and trajectory group membership, a total of 6 logistic regression models were computed (for full tables, see Additional File A1 Tables S12-17), accounting for a maximum of 1 predictor per 15 participants belonging to the smallest outcome category. Few statistically significant associations were found. For sleep duration trajectories, in the age and gender model, we found that participants who reported being 25 years or older were consistently less likely to belong to the long and decreasing (Age groups 25-34 : [OR] =0.35; 95%CI = 0.18 – 0.70; p = .0028, age groups 35-44 [OR] =0.30; 95%CI = 0.15 – 0.60; p =.0005, age groups 45-54 [OR] =0.28; 95%CI = 0.14 – 0.53; p =.0001, age groups 55-64 [OR] =0.34; 95%CI = 0.19 – 0.63; p = .0007, age group 65+ [OR] =0.41; 95%CI = 0.22 – 0.76; p = 0.0043) sleep duration trajectory group than participants who reported being 18 to 25 years old. In the model containing all other predictors, participants who reported a household size greater than one were 2.57 times more likely to belong to the long and decreasing sleep duration trajectory group ([OR] = 2.57; 95%CI = 1.43 – 4.60; p = .0016), than participants who reported a household size of one.
 Regarding sleep quality trajectories, in the age and gender model, participants who reported being aged 65 years or older were 7.11 times more likely to belong to the very high and stable ([OR] =7.11; 95%CI = 1.38 – 36.57; p = .0188) sleep quality trajectory group than participants reported being 18 to 25 years old. Additionally, participants who identified as male were 2.17 times more likely to belong to the high and stable ([OR] =2.17; 95%CI = 1.15 – 4.10; p = .0165) sleep quality trajectory group than participants who identified as female. In the model containing all other predictors, participants who reported following the government’s recommendations were 1.41 and 2.02 times more likely to belong to the high and stable ([OR] =1.41; 95%CI = 1.01 – 1.96; p =.0429) and very high and stable sleep quality trajectory groups ([OR] =2.02; 95%CI = 1.28 – 3.17; p =.0024), respectively, than participants who did not. In the ethnic background model, the predictor was not statistically significant.

## Joint Trajectories Analysis of Sleep Quality and Sleep Duration during the First Wave of the COVID-19 Pandemic

The joint trajectory analysis results (see Table 6) indicate that the likelihood of belonging to the long and decreasing sleep duration trajectory group for those who belonged to the very high and stable sleep quality trajectory group was statistically significant at 98.9% (p < .001). No other associations were significant.

Table 6

*Joint Trajectory Analysis Results*

| Group | Low and Stable Sleep Quality | Moderate and Stable Sleep Quality | High and Stable Sleep Quality | Very High and Stable Sleep Quality |
| --- | --- | --- | --- | --- |
|  |  |  |  |  |
|  | Probability of Sleep Quality Group Conditional on Sleep Duration Group | | | |
|  |  | | | |
| Short and Stable Sleep Duration | .220 | .659 | .118 | .003 |
|  |  |  |  |  |
| Long and Decreasing Sleep Duration | .009 | .214 | .619 | .159 |
|  |  |  |  |  |
|  | Probability of Sleep Duration Group Conditional on Sleep Quality Group | | | |
|  |  | | | |
| Short and Stable Sleep Duration | .928 | .612 | .089 | .011 |
|  |  |  |  |  |
| Long and Decreasing Sleep Duration | .072 | .388 | .911 | **.989***** |
|  |  |  |  |  |

*Note.* *** p-value < 1e-3.

# Discussion

In our study, we sought to understand the unique impact of COVID-19 on health by examining the intraindividual variations in sleep quality and sleep duration over time during the first wave of the pandemic in the Canadian adult population and exploring which individual characteristics predict unstable sleep. A handful of Canadian studies previously reported a decrease in sleep quality and duration, but none of those studies measured sleep longitudinally throughout the first wave of the pandemic and were representative of the population. In contrast, our study uses a longitudinal design and a representative sample. Our results point to a very small decrease in sleep duration, which was limited to two-thirds of our sample, and no changes in sleep quality throughout the first wave of COVID-19. Furthermore, younger age and living with someone predicted a greater likelihood of belonging to an unstable sleep duration trajectory.

Overall, our results align with findings from other Canadian studies rather than those of studies conducted abroad, pointing toward a general decrease in sleep duration rather than an increase, albeit also to a stable sleep quality instead of a decrease. The observed stability of sleep quality differed from the findings reported in Morin and colleagues’ study, ^19^ which reported a sleep quality decrease during the first COVID-19 wave (April – May 2020) compared with 2017-2018 prepandemic data. However, their sample was subject to a selection bias, as it consisted mostly of residents from the province of Quebec, where additional COVID-19 policies were implemented, such as travel restrictions and non-essential business closures, ^27,28^ likely increasing the burden of COVID-19 on daily life, and a high proportion of insomniacs, who are likely to report worse sleep. They reported a decrease in sleep quality during the first wave of the pandemic compared with prepandemic times. An increase in the emergence of sleep difficulties during the first COVID-19 wave (April 3 – June 24) in comparison with preoutbreak times (1 month before the outbreak) was measured retrospectively by Robillard et al. ^25^ This finding was also observed in other studies conducted worldwide using retrospective sleep measurement tools referring to different prepandemic times. ^13,20,73^ However, retrospective measures may overestimate the impact of the pandemic on sleep as they introduce recall bias. Alternatively, in our study, we observed exclusively stable sleep quality trajectory groups.

Instead of an overall increase in sleep duration during the first wave of the pandemic in comparison with prepandemic data as reported abroad, ^13,16,17,74^ we observed that sleep in Canada was mostly stable for one-third of our sample and decreased slightly for the other two-thirds. A study by Carroll and colleagues ^26^ conducted on Canadian families during the first COVID-19 wave also found that sleep was mostly stable in approximately half of their sample and decreased in the other third between the start of the COVID-19 pandemic (“since COVID-19”) and April–May 2020. However, their findings were limited by the retrospective nature of their prepandemic data and limited to the province of Ontario, which implemented additional non-essential business closure policies unseen in most provinces of Canada. ^28^ Robillard et al. ^25^ also reported a decrease in sleep duration during the first COVID-19 wave (April 3 – June 24) in comparison with preoutbreak times (1 month before the outbreak), although they did not report whether this decrease was led by a subgroup of participants or across their entire sample. Our results extend the findings of Robillard et al. and Carroll et al.’s works, as they provide a more complete understanding of the two different trajectories of sleep duration reported by the public during the first wave of the pandemic. This discrepancy between local and foreign studies regarding sleep duration changes could be due to the retrospective nature of prior studies, which might reflect erroneous location-specific participant beliefs about the effects of COVID-19 on their sleep. Furthermore, our results support that being older than 25 years predicts a stable sleep duration trajectory, which is in line with previous studies conducted worldwide during the first COVID-19 wave, which reported that old age is a protective factor for sleep health. ^29,32,33^ Nevertheless, contrary to what was hypothesized, living alone or with minors was not shown to predict an unstable sleep duration trajectory. The results from Morin and colleagues’ study ^19^ instead support that living alone was associated with increased fatigue and that lower social support was associated with more severe insomnia and poorer sleep during the first wave of the pandemic. This discrepancy between these findings may be explained by the generalizability of their results, as their data were obtained from a larger study designed to investigate a sample of individuals with insomnia, which may be subject to selection bias.

Our results suggest that sleep is overall stable in the context of the COVID-19 crisis, suggesting that sleep was more likely governed by factors that remained stable throughout the pandemic, such as environmental signals that entrain circadian rhythms (i.e., zeitgebers), such as light exposure, ^75^ meal timing, ^76^ and some aspects of social routines, ^77^ such as online interactions and in-person household interactions, rather than other contextual environmental factors, such as being confined to one’s home. The remarkably small number of significant predictors of unstable sleep that we observed could reflect the stabilizing power of these factors. Subsequent research could investigate the underlying causes of the overall stability of sleep that was observed during the first wave of the pandemic. The heterogeneity between the two sleep duration trajectory groups (one stable, the other unstable) may be explained by the competing effects of home confinement or remotely performed activities. As suggested by Altena et al. (2020), ^78^ home confinement can likely be detrimental to sleep duration since it forces individuals to commit to various activities that might deprive them of sleep time, such as house administration, homeschooling, and household errands, and burdening them with additional stress. However, home confinement can also be beneficial to sleep, as time normally spent at work and in transit to work is likely reduced due to teleworking and business closures, freeing more time for sleeping but also for engaging in healthy behaviors beneficial to sleep and allowing more freedom to sleep according to one’s chronotype. ^78^ The addition of a measure of subjective freedom to sleep would allow a better understanding of this sleep duration disparity. The stability of sleep may also be linked to the characteristics of the population most at risk from the viral threat and the public health measures. During the pandemic, there was a notable decline in health services particularly for residents of long-term care facilities, who represented 67% of COVID-19-related deaths in Canada as of February 15, 2021. ^79^ In contrast, individuals living outside long-term care facilities may not have faced the same disruptive changes to their quality of life, as they resided outside of these restrictive environments.
Our study revealed several factors that were protective of sleep duration stability. The first is older age, which has been suggested to increase resilience to crises due to the slower pace of life and reduced social and economic pressure that accompanies an old age lifestyle, along with the use of past coping skills acquired from life experiences to address fear and uncertainty. ^80^ Some studies support that having a sense of being able to successfully adapt to challenging experiences (resilience) is a potential buffering factor against sleep disturbances and poor sleep quality. ^81,82^ Belonging to the oldest age group (65+) was also associated with reporting the highest sleep quality, further supporting the protective role of age on sleep.
The second factor was living alone, which was not associated with a greater likelihood of an unstable sleep duration trajectory. Living with someone might reflect the presence of family responsibilities associated with the emergence of new sleep difficulties during the first wave of the pandemic. Family responsibilities might increase the likelihood of the occurrence of relational conflicts, which are reported to be rising worldwide during the pandemic, ^83^ and bed-sharing, both of which are associated with poorer sleep outcomes. ^84–86^ Living alone might also decrease the risk of contracting COVID-19 through household transmission and alleviate pandemic-related life disruptions. Living alone might also not have impacted social life significantly during COVID-19, as social connectedness rose during the pandemic. ^87^ The addition of a measure of family responsibilities would shed light upon the mechanism driving the protective effect of living alone on sleep.
In contrast, those in the long and unstable sleep duration group were more likely to belong to the highest sleep quality trajectory group. This might be attributable to the reported reduction in changes in daily routines between work days and free days due to the implementation of stay-at-home orders and the transition to telework during the first wave of the pandemic. ^88,89^ Sleep tends to be insufficient during work days or constrained to a schedule that is maladapted to an individual’s chronotype, leading to an accumulation of sleep debt, but is usually recovered during free days, as sleep is allowed to last longer and schedules are looser, a phenomenon known as social jet lag. ^90^ Accordingly, COVID-19-related social restrictions might have reduced social jet lag by allowing more freedom to sleep according to one’s needs, thus allowing them to repay their sleep debt and perceive a higher quality of sleep. As sleep debt is repaid, sleep duration decreases over time. This is known as sleep satiation. Having access to prepandemic data for comparison with pandemic data would allow researchers to verify that hypothesis by revealing whether this decrease in sleep duration followed a sudden increase in sleep duration following the earliest pandemic disruptions.
The results also highlight that ethnicity, overall, was not a predictor of deficient sleep. Our study has several strengths. Our study was the first to collect sleep data throughout the first wave of the pandemic on a biweekly basis throughout Canada, a critical time that was marked by the early days of the pandemic. Our study was also the first to recruit a representative sample from Canada through a quota-based sampling method. This sample reflected the adult Canadian population in terms of age, gender identity, province of residence, household size, current occupation, and country of origin. ^55^ Our analysis also reveals that our final sample was representative in terms of ethnicity (see Table 3). Additionally, identifiable sociological and demographic variations in our sample were also mitigated through a weighting process using census data from Statistics Canada, ^57,64^ based on the presence of household members under 18 years old, on the province of residence, and aboriginal background, improving the representativeness of our sample. Moreover, this study is also the first longitudinal study to survey sleep more than twice in a Canadian sample during the first wave of COVID-19. Finally, our study was the first to use latent class growth analysis to detect potential sleep duration and quality trajectories during the first wave of the pandemic throughout Canada. This methodology allowed us to determine subtle patterns of change in sleep outcomes over time and to distinguish subgroups following different patterns of change over time, when applicable.

Our study has several limitations. The use of sleep quality and sleep duration items based on the last 24 hours may not have been representative of the last two weeks; most commonly validated sleep questionnaires tend to assess a period between one week and 4 weeks. ^91^ However, there were no differences between weekday and weekend responders, and Levene’s test indicated that there were no significant differences in the variances between weekday and weekend responders. These results suggest that the day of the week most likely did not significantly impact the survey results. Furthermore, our findings may not apply to all populations, as they excluded individuals without internet access and those who could not understand English or French. However, these populations are estimated to represent 6% ^92^ and 1.8%, ^93^ respectively, of the Canadian population. Additionally, despite being representative of the Canadian population in terms of age, gender identity, province of residence, ethnicity, household size, current occupation, and country of origin, our final sample may not have been representative of other sociological and demographic variables. Of note, as reported previously, ^55^ Canadians with lower levels of education (for instance, 45.7% without bachelor’s degree in sample, 71.5% in Census data), native French speakers (Sample 16.6%, Census 21,1%), and Indigenous people (Sample: 2.7%; Census 4.9%) were underrepresented in our original sample according to the Statistics Canada 2016 census profile. We attempted to mitigate this limitation by using a weighting process to correct identifiable sociological and demographic imbalances, utilizing data from 2016 from Statistics Canada. ^57^ However, use of quota-based non-probabilistic sampling may increase unmeasured heterogeneity into our sample, and thus potentially impact our results. The use of a weighting technique to address disparities between our sample and the Canadian population may not reduce selection bias for variables that were weakly associated with sociological and demographic factors, thus affecting the generalizability of our findings. As shown by Haddad et al., ^94^ the use of weighting to correct for sociological and demographic features of the sample may not necessarily change results. Taken together, it is therefore possible that differences between the original sample and the Canadian population may have persisted, decreasing the generalizability of our findings. Furthermore, since participants were required to be Canadian citizens or permanent residents, our study does not include temporary workers, who were estimated to represent approximately 2.9% of the population in 2017. ^95^ However, this proportion might have been reduced during the first wave of the pandemic due to travel restrictions and a sudden slowdown in economic activity. Mental health problems that impact sleep, such as depression or Post-Traumatic Stress Disorder, were also not measured, as our data collection means (online questionnaires) did not allow us to collect reliable data. Finally, the findings in this study are possibly limited to crises similar in nature and scale to the COVID-19 pandemic, and may not extend to other crises, such as individual-level crises.

# Conclusion

This study is the first to provide empirical evidence on sleep trajectories and their health-related predictors during the first wave of the COVID-19 pandemic utilizing a large representative sample of Canadians. Our findings that sleep duration and sleep quality remained stable overall throughout the first 4 months of the pandemic support the notion that sleep is governed by a constellation of factors that lead to stable and resilient sleep, even in the context of a crisis. Similarly, we found that people living with others were more likely to report longer sleep durations and a slight decrease in sleep duration over time. Furthermore, adults aged 25 years or older were less likely to belong to the unstable (longer sleep) trajectory. Future studies should examine whether similar results are observed in more diverse samples.

# Declarations

## Ethics approval and consent to participate

The IRB approval for this study was given by the Research Ethics Committee in Education and Psychology of the University of Montreal. ^55^ Informed consent was obtained for experimentation with human subjects.

## Consent for publication

Not applicable.

## Availability of data and materials

Unfortunately, the dataset used and analyzed during the current study is not available, as we did not receive ethical approval to make the data available.

## Competing interests

The authors declare that they have no competing interests.

## Funding

This work was supported by the Canadian Institutes of Health Research (CIHR) [grant number 170633], the Social Sciences and Humanities Research Council (SSHRC), and the Centre for the Study of Democratic Citizenship (CSDC). Additionally, GS was supported by an FRQS Research Scholar award and financial support from the Research Center of the CIUSSS NIM.

## Authors' contributions

All the authors contributed to the study conception and design, commented on previous versions of the manuscript, and read and approved the final manuscript. AL also wrote the manuscript, performed the analyses, and created the published work. MPD contributed to the investigation, to methodology, to statistical analyses, to data curation, and to the validation of the results. RdlS provided study resources and led the investigation, funding acquisition, and project planning. GS also led project supervision.

## Acknowledgements

We would like to thank everyone involved in this project for their contribution. Special thanks are given to Clémentine Courdi, research counselor, for constructing custom trajectory model graphics, and to Prof. Bobby Jones for guiding the usage of his SAS PROC TRAJ package used in this study. We also thank all the research participants for their time and effort in completing the questionnaire multiple times in a time of need.

# References

1. Gao C, Guo J, Gong TT, et al. Sleep Duration/Quality With Health Outcomes: An Umbrella Review of Meta-Analyses of Prospective Studies. *Front Med*. 2022;8. doi:10.3389/fmed.2021.813943

2. Scott AJ, Webb TL, Martyn-St James M, Rowse G, Weich S. Improving sleep quality leads to better mental health: A meta-analysis of randomised controlled trials. *Sleep Medicine Reviews*. 2021;60:101556. doi:10.1016/j.smrv.2021.101556

3. Liu RT, Steele SJ, Hamilton JL, et al. Sleep and suicide: A systematic review and meta-analysis of longitudinal studies. *Clinical Psychology Review*. 2020;81:101895. doi:10.1016/j.cpr.2020.101895

4. Winsper C, Tang NKY. Linkages between insomnia and suicidality: Prospective associations, high-risk subgroups and possible psychological mechanisms. *International Review of Psychiatry*. 2014;26(2):189-204. doi:10.3109/09540261.2014.881330

5. Neckelmann D, Mykletun A, Dahl AA. Chronic Insomnia as a Risk Factor for Developing Anxiety and Depression. *Sleep*. 2007;30(7):873-880. doi:10.1093/sleep/30.7.873

6. Pigeon WR, Bishop TM, Krueger KM. Insomnia as a Precipitating Factor in New Onset Mental Illness: a Systematic Review of Recent Findings. *Curr Psychiatry Rep*. 2017;19(8):44. doi:10.1007/s11920-017-0802-x

7. Hertenstein E, Feige B, Gmeiner T, et al. Insomnia as a predictor of mental disorders: A systematic review and meta-analysis. *Sleep Medicine Reviews*. 2019;43:96-105. doi:10.1016/j.smrv.2018.10.006

8. Li L, Wu C, Gan Y, Qu X, Lu Z. Insomnia and the risk of depression: a meta-analysis of prospective cohort studies. *BMC Psychiatry*. 2016;16(1):375. doi:10.1186/s12888-016-1075-3

9. Wu L, Sun D, Tan Y. A systematic review and dose-response meta-analysis of sleep duration and the occurrence of cognitive disorders. *Sleep Breath*. 2018;22(3):805-814. doi:10.1007/s11325-017-1527-0

10. Medic G, Wille M, Hemels ME. Short- and long-term health consequences of sleep disruption. *Nature and Science of Sleep*. 2017;9:151-161. doi:10.2147/NSS.S134864

11. Public Health Agency of Canada. Are Canadian adults getting enough sleep? September 6, 2019. Accessed May 10, 2023. https://www.canada.ca/en/public-health/services/publications/healthy-living/canadian-adults-getting-enough-sleep-infographic.html

12. de la Sablonnière R. Toward a Psychology of Social Change: A Typology of Social Change. *Front Psychol*. 2017;8. doi:10.3389/fpsyg.2017.00397

13. Neculicioiu VS, Colosi IA, Costache C, Sevastre-Berghian A, Clichici S. Time to Sleep?—A Review of the Impact of the COVID-19 Pandemic on Sleep and Mental Health. *Int J Environ Res Public Health*. 2022;19(6):3497. doi:10.3390/ijerph19063497

14. Trabelsi K, Ammar A, Masmoudi L, et al. Sleep Quality and Physical Activity as Predictors of Mental Wellbeing Variance in Older Adults during COVID-19 Lockdown: ECLB COVID-19 International Online Survey. *International Journal of Environmental Research and Public Health*. 2021;18(8):4329. doi:10.3390/ijerph18084329

15. Trakada A, Nikolaidis PT, Andrade M dos S, et al. Sleep During “Lockdown” in the COVID-19 Pandemic. *International Journal of Environmental Research and Public Health*. 2020;17(23):9094. doi:10.3390/ijerph17239094

16. Blume C, Schmidt MH, Cajochen C. Effects of the COVID-19 lockdown on human sleep and rest-activity rhythms. *Current Biology*. 2020;30(14):R795-R797. doi:10.1016/j.cub.2020.06.021

17. Bottary R, Fields EC, Kensinger EA, Cunningham TJ. Age and chronotype influenced sleep timing changes during the first wave of the COVID-19 pandemic. *Journal of Sleep Research*. 2022;31(2):e13495. doi:10.1111/jsr.13495

18. Marelli S, Castelnuovo A, Somma A, et al. Impact of COVID-19 lockdown on sleep quality in university students and administration staff. *J Neurol*. 2021;268(1):8-15. doi:10.1007/s00415-020-10056-6

19. Morin CM, Vézina-Im LA, Ivers H, et al. Prevalent, incident, and persistent insomnia in a population-based cohort tested before (2018) and during the first-wave of COVID-19 pandemic (2020). *Sleep*. 2021;45(1):zsab258. doi:10.1093/sleep/zsab258

20. French MT, Mortensen K, Timming AR. Changes in self-reported health, alcohol consumption, and sleep quality during the COVID-19 pandemic in the United States. *Applied Economics Letters*. 2022;29(3):219-225. doi:10.1080/13504851.2020.1861197

21. Simonelli G, Petit D, Delage JP, et al. Sleep in times of crises: A scoping review in the early days of the COVID-19 crisis. *Sleep Medicine Reviews*. 2021;60:101545. doi:10.1016/j.smrv.2021.101545

22. Meaklim H, Le F, Drummond SPA, et al. Insomnia is more likely to persist than remit after a time of stress and uncertainty: a longitudinal cohort study examining trajectories and predictors of insomnia symptoms. *Sleep*. 2024;47(4):zsae028. doi:10.1093/sleep/zsae028

23. Rutty CJ. COVID-19 Pandemic in Canada. In: *The Canadian Encyclopedia*. ; 2023. Accessed June 18, 2023. https://www.thecanadianencyclopedia.ca/en/article/covid-19-pandemic

24. Detsky AS, Bogoch II. COVID-19 in Canada: Experience and Response to Waves 2 and 3. *JAMA*. 2021;326(12):1145-1146. doi:10.1001/jama.2021.14797

25. Robillard R, Dion K, Pennestri MH, et al. Profiles of sleep changes during the COVID-19 pandemic: Demographic, behavioural and psychological factors. *Journal of Sleep Research*. 2021;30(1):e13231. doi:10.1111/jsr.13231

26. Carroll N, Sadowski A, Laila A, et al. The Impact of COVID-19 on Health Behavior, Stress, Financial and Food Security among Middle to High Income Canadian Families with Young Children. *Nutrients*. 2020;12(8):2352. doi:10.3390/nu12082352

27. Joëlle Girard. Des barrages routiers limitent l’accès à huit régions du Québec. *Radio-Canada*. https://ici.radio-canada.ca/nouvelle/1689260/coronavirus-quebec-bilan-morts-montreal-etat-urgence. March 28, 2020. Accessed September 29, 2024.

28. Tyler Dawson. COVID-19: Ontario and Quebec order non-essential businesses closed after spike in coronavirus totals. *National Post*. https://nationalpost.com/news/covid-19-ontario-and-quebec-order-non-essential-businesses-closed-after-spike-in-coronavirus-totals. March 23, 2020. Accessed September 29, 2024.

29. Desai B, Carrigan N, Wearn A, et al. 32 Sleep quality, mental health, and circadian rhythms during COVID lockdown – results from the SleepQuest study. *BMJ Open Respiratory Research*. 2021;8(Suppl 1). doi:10.1136/bmjresp-2021-bssconf.29

30. Casagrande M, Favieri F, Tambelli R, Forte G. The enemy who sealed the world: effects quarantine due to the COVID-19 on sleep quality, anxiety, and psychological distress in the Italian population. *Sleep Med*. 2020;75:12-20. doi:10.1016/j.sleep.2020.05.011

31. Ramos Socarras L, Potvin J, Forest G. COVID-19 and sleep patterns in adolescents and young adults. *Sleep Med*. 2021;83:26-33. doi:10.1016/j.sleep.2021.04.010

32. Wester CT, Bovil T, Scheel-Hincke LL, Ahrenfeldt LJ, Möller S, Andersen-Ranberg K. Longitudinal changes in mental health following the COVID-19 lockdown: Results from the Survey of Health, Ageing, and Retirement in Europe. *Ann Epidemiol*. 2022;74:21-30. doi:10.1016/j.annepidem.2022.05.010

33. Yuksel D, McKee GB, Perrin PB, et al. Sleeping when the world locks down: Correlates of sleep health during the COVID-19 pandemic across 59 countries. *Sleep Health: Journal of the National Sleep Foundation*. 2021;7(2):134-142. doi:10.1016/j.sleh.2020.12.008

34. Gong K, Garneau J, Grenier S, et al. Insomnia symptoms among older adults during the first year of the COVID-19 pandemic: A longitudinal study. *Sleep Health*. 2023;9(4):560-566. doi:10.1016/j.sleh.2023.04.008

35. Kowall SM, Sommer JL, Reynolds KA, Mota N, El-Gabalawy R. Sleep disturbance during COVID-19: Correlates and predictive ability for mental health symptomatology in a Canadian online sample. *Gen Hosp Psychiatry*. 2023;80:48-53. doi:10.1016/j.genhosppsych.2023.01.002

36. Bann D, Villadsen A, Maddock J, et al. Changes in the behavioural determinants of health during the COVID-19 pandemic: gender, socioeconomic and ethnic inequalities in five British cohort studies. *J Epidemiol Community Health*. 2021;75(12):1136-1142. doi:10.1136/jech-2020-215664

37. Salfi F, Lauriola M, Amicucci G, et al. Gender-related time course of sleep disturbances and psychological symptoms during the COVID-19 lockdown: A longitudinal study on the Italian population. *Neurobiology of Stress*. 2020;13:100259. doi:10.1016/j.ynstr.2020.100259

38. Taporoski TP, Beijamini F, Gómez LM, et al. Subjective sleep quality before and during the COVID-19 pandemic in a Brazilian rural population. *Sleep Health: Journal of the National Sleep Foundation*. 2022;8(2):167-174. doi:10.1016/j.sleh.2021.11.007

39. Falkingham J, Evandrou M, Qin M, Vlachantoni A. “Sleepless in Lockdown”: unpacking differences in sleep loss during the coronavirus pandemic in the UK. Published online July 21, 2020:2020.07.19.20157255. doi:10.1101/2020.07.19.20157255

40. Salfi F, D’Atri A, Tempesta D, Ferrara M. Sleeping under the waves: A longitudinal study across the contagion peaks of the COVID-19 pandemic in Italy. *Journal of Sleep Research*. 2021;30(5):e13313. doi:10.1111/jsr.13313

41. Shillington KJ, Vanderloo LM, Burke SM, Ng V, Tucker P, Irwin JD. Not so sweet dreams: adults’ quantity, quality, and disruptions of sleep during the initial stages of the COVID-19 pandemic. *Sleep Med*. 2022;91:189-195. doi:10.1016/j.sleep.2021.02.028

42. Martínez-de-Quel Ó, Suárez-Iglesias D, López-Flores M, Pérez CA. Physical activity, dietary habits and sleep quality before and during COVID-19 lockdown: A longitudinal study. *Appetite*. 2021;158:105019. doi:10.1016/j.appet.2020.105019

43. Shi L, Lu ZA, Que JY, et al. Prevalence of and Risk Factors Associated With Mental Health Symptoms Among the General Population in China During the Coronavirus Disease 2019 Pandemic. *JAMA Netw Open*. 2020;3(7):e2014053. doi:10.1001/jamanetworkopen.2020.14053

44. Lin L yu, Wang J, Ou-yang X yong, et al. The immediate impact of the 2019 novel coronavirus (COVID-19) outbreak on subjective sleep status. *Sleep Medicine*. 2021;77:348-354. doi:10.1016/j.sleep.2020.05.018

45. Salari N, Khazaie H, Hosseinian-Far A, et al. The prevalence of sleep disturbances among physicians and nurses facing the COVID-19 patients: a systematic review and meta-analysis. *Global Health*. 2020;16(1):92. doi:10.1186/s12992-020-00620-0

46. Ballesio A, Zagaria A, Musetti A, et al. Longitudinal associations between stress and sleep disturbances during COVID-19. *Stress and Health*. 2022;38(5):919-926. doi:10.1002/smi.3144

47. Jahrami H, BaHammam AS, Bragazzi NL, Saif Z, Faris M, Vitiello MV. Sleep problems during the COVID-19 pandemic by population: a systematic review and meta-analysis. *Journal of Clinical Sleep Medicine*. 2021;17(2):299-313. doi:10.5664/jcsm.8930

48. Cheng P, Casement MD, Cuellar R, et al. Sleepless in COVID-19: racial disparities during the pandemic as a consequence of structural inequity. *Sleep*. 2022;45(1):zsab242. doi:10.1093/sleep/zsab242

49. Government of Canada SC. Visible Minority and Population Group Reference Guide, Census of Population, 2016. 2017. Accessed September 27, 2024. https://www12.statcan.gc.ca/census-recensement/2016/ref/guides/006/98-500-x2016006-eng.cfm

50. Power T, Wilson D, Best O, et al. COVID-19 and Indigenous Peoples: An imperative for action. *Journal of Clinical Nursing*. 2020;29(15-16):2737-2741. doi:10.1111/jocn.15320

51. Speers D. Role of testing for the COVID-19 response in Aboriginal, Torres Strait Islander and regional populations. *Microbiol Aust*. 2021;42(1):23-26. doi:10.1071/MA21007

52. Department of Justice of Canada. Government of Canada’s response to COVID-19. September 21, 2023. Accessed January 10, 2023. https://justice.gc.ca/eng/csj-sjc/covid.html

53. Buysse DJ. Sleep Health: Can We Define It? Does It Matter? *Sleep*. 2014;37(1):9-17. doi:10.5665/sleep.3298

54. Bin YS. Is Sleep Quality More Important than Sleep Duration for Public Health? *Sleep*. 2016;39(9):1629-1630. doi:10.5665/sleep.6078

55. de la Sablonnière R, Dorfman A, Pelletier-Dumas M, et al. *COVID-19 Canada: The end of the world as we know it? (Technical report No. 1). Presenting the COVID-19 Survey.* Université de Montréal; 2020. Accessed March 2, 2023. https://a5eaedf4-8d49-4b28-9cae-77db17679ab5.filesusr.com/ugd/68376a_e857b040c5ac480997b7ce002ac546bc.pdf

56. Delvinia Holdings Inc. Delvinia. Accessed October 22, 2023. https://www.delvinia.com/solutions/askingcanadians/

57. Statistics Canada. Census Profile, 2016 Census. Published online February 8, 2017. Accessed September 27, 2023. https://www12.statcan.gc.ca/census-recensement/2016/dp-pd/prof/index.cfm?Lang=E

58. Johnston R, Brady HE. The rolling cross-section design. *Electoral Studies*. 2002;21(2):283-295. doi:10.1016/S0261-3794(01)00022-1

59. Caron-Diotte M, Dorfman A, Pelletier-Dumas M, et al. *COVID-19 Canada: The end of the world as we know it? Technical Report No. 2. Handling planned and unplanned missing data*.; 2021. Accessed June 2, 2023. https://csdc-cecd.wixsite.com/covid19csi/resultats?lang=en

60. Rhemtulla M, Little T. Tools of the Trade: Planned Missing Data Designs for Research in Cognitive Development. *J Cogn Dev*. 2012;13(4). doi:10.1080/15248372.2012.717340

61. Enders CK. *Applied Missing Data Analysis*. 2nd ed. Guilford Publications; 2022:xv, 377. Accessed June 2, 2023. https://books.google.ca/books?id=uHt4EAAAQBAJ&lpg=PP1&ots=W0sNJ6tCQc&dq=Applied%20missing%20data%20analysis%20Enders%20Craig&lr&pg=PP1#v=onepage&q=Applied%20missing%20data%20analysis%20Enders%20Craig&f=false

62. Public Health Agency of Canada. COVID-19 daily epidemiology update: Current situation. April 19, 2020. Accessed April 16, 2023. https://health-infobase.canada.ca/covid-19/current-situation.html

63. Mercer A, Lau A, Kennedy C. For Weighting Online Opt-In Samples, What Matters Most? Published online January 26, 2018. Accessed March 13, 2023. https://policycommons.net/artifacts/617484/for-weighting-online-opt-in-samples-what-matters-most/1598296/

64. Statistics Canada. *Table 17-10-0005-01 Population Estimates on July 1st, by Age and Sex*.; 2022. Accessed March 28, 2023. https://doi.org/10.25318/1710000501-eng

65. National Sleep Foundation. What Is Sleep Quality? National Sleep Foundation. April 12, 2024. Accessed September 29, 2024. https://www.thensf.org/what-is-sleep-quality/

66. Buysse DJ, Reynolds CF, Monk TH, Berman SR, Kupfer DJ. The Pittsburgh sleep quality index: A new instrument for psychiatric practice and research. *Psychiatry Research*. 1989;28(2):193-213. doi:10.1016/0165-1781(89)90047-4

67. Government of Canada. National Occupational Classification. National Occupational Classification. 2021. Accessed June 1, 2023. https://noc.esdc.gc.ca/?GoCTemplateCulture=en-CA

68. Jones BL, Nagin DS, Roeder K. A SAS Procedure Based on Mixture Models for Estimating Developmental Trajectories. *Sociological Methods & Research*. 2001;29(3):374-393. doi:10.1177/0049124101029003005

69. Jones BL, Nagin DS. Advances in Group-Based Trajectory Modeling and an SAS Procedure for Estimating Them. *Sociological Methods & Research*. 2007;35(4):542-571. doi:10.1177/0049124106292364

70. Nagin DS. Analyzing developmental trajectories: A semiparametric, group-based approach. *Psychological Methods*. 1999;4:139-157. doi:10.1037/1082-989X.4.2.139

71. Frankfurt S, Frazier P, Syed M, Jung KR. Using Group-Based Trajectory and Growth Mixture Modeling to Identify Classes of Change Trajectories. *The Counseling Psychologist*. 2016;44(5):622-660. doi:10.1177/0011000016658097

72. D’Unger A, Land KC, McCall PL, Nagin DS. How Many Latent Classes of Delinquent/Criminal Careers? Results from Mixed Poisson Regression Analyses. *American Journal of Sociology*. 1998;103(6):1593-1630. doi:10.1086/231402

73. Limongi F, Siviero P, Trevisan C, et al. Changes in sleep quality and sleep disturbances in the general population from before to during the COVID-19 lockdown: A systematic review and meta-analysis. *Frontiers in Psychiatry*. 2023;14. doi:10.3389/fpsyt.2023.1166815

74. Rezaei N, Grandner MA. Changes in sleep duration, timing, and variability during the COVID-19 pandemic: Large-scale Fitbit data from 6 major US cities. *Sleep Health*. 2021;7(3):303-313. doi:10.1016/j.sleh.2021.02.008

75. Roenneberg T, Daan S, Merrow M. The art of entrainment. *J Biol Rhythms*. 2003;18(3):183-194. doi:10.1177/0748730403018003001

76. Banks S, Dorrian J, Grant C, Coates A. Chapter 17 - Circadian Misalignment and Metabolic Consequences: Shiftwork and Altered Meal Times. In: Watson RR, ed. *Modulation of Sleep by Obesity, Diabetes, Age, and Diet*. Academic Press; 2015:155-164. doi:10.1016/B978-0-12-420168-2.00017-X

77. Moss TG, Carney CE, Haynes P, Harris AL. Is daily routine important for sleep? An investigation of social rhythms in a clinical insomnia population. *Chronobiology International*. 2015;32(1):92-102. doi:10.3109/07420528.2014.956361

78. Altena E, Baglioni C, Espie CA, et al. Dealing with sleep problems during home confinement due to the COVID-19 outbreak: Practical recommendations from a task force of the European CBT-I Academy. *Journal of Sleep Research*. 2020;29(4):e13052. doi:10.1111/jsr.13052

79. Betini R, Lawand SM and C. The Impact of the COVID-19 Pandemic in Long-Term Care in Canada. *Healthcare Quarterly*. 2021;24(3). Accessed October 29, 2024. https://www.longwoods.com/content/26625/healthcare-quarterly/the-impact-of-the-covid-19-pandemic-in-long-term-care-in-canada

80. McKinlay AR, Fancourt D, Burton A. A qualitative study about the mental health and wellbeing of older adults in the UK during the COVID-19 pandemic. *BMC Geriatrics*. 2021;21(1):439. doi:10.1186/s12877-021-02367-8

81. Liu X, Liu C, Tian X, et al. Associations of Perceived Stress, Resilience and Social Support with Sleep Disturbance Among Community-dwelling Adults. *Stress and Health*. 2016;32(5):578-586. doi:10.1002/smi.2664

82. Downing MJ, Houang ST, Scheinmann R, Yoon IS, Chiasson MA, Hirshfield S. Engagement in care, psychological distress, and resilience are associated with sleep quality among HIV-positive gay, bisexual, and other men who have sex with men. *Sleep Health*. 2016;2(4):322-329. doi:10.1016/j.sleh.2016.08.002

83. Campbell AM. An increasing risk of family violence during the Covid-19 pandemic: Strengthening community collaborations to save lives. *Forensic Science International: Reports*. 2020;2:100089. doi:10.1016/j.fsir.2020.100089

84. Andre CJ, Lovallo V, Spencer RMC. The effects of bed sharing on sleep: From partners to pets. *Sleep Health*. 2021;7(3):314-323. doi:10.1016/j.sleh.2020.11.011

85. Gordon AM, Carrillo B, Barnes CM. Sleep and social relationships in healthy populations: A systematic review. *Sleep Medicine Reviews*. 2021;57:101428. doi:10.1016/j.smrv.2021.101428

86. Liu X, Liu L, Wang R. Bed Sharing, Sleep Habits, and Sleep Problems Among Chinese School-Aged Children. *Sleep*. 2003;26(7):839-844. doi:10.1093/sleep/26.7.839

87. Pandya A, Lodha P. Social Connectedness, Excessive Screen Time During COVID-19 and Mental Health: A Review of Current Evidence. *Front Hum Dyn*. 2021;3. doi:10.3389/fhumd.2021.684137

88. Korman M, Tkachev V, Reis C, et al. COVID-19-mandated social restrictions unveil the impact of social time pressure on sleep and body clock. *Sci Rep*. 2020;10(1):22225. doi:10.1038/s41598-020-79299-7

89. Leone MJ, Sigman M, Golombek DA. Effects of lockdown on human sleep and chronotype during the COVID-19 pandemic. *Current Biology*. 2020;30(16):R930-R931. doi:10.1016/j.cub.2020.07.015

90. Wittmann M, Dinich J, Merrow M, Roenneberg T. Social Jetlag: Misalignment of Biological and Social Time. *Chronobiology International*. 2006;23(1-2):497-509. doi:10.1080/07420520500545979

91. Redline S, Redline B, James P. Sleep Epidemiology: An Introduction. In: Duncan DT, Kawachi I, Redline S, eds. *The Social Epidemiology of Sleep*. Oxford University Press; 2019:11-C2.P200. doi:10.1093/oso/9780190930448.003.0002

92. Statistics Canada. *Access to the Internet in Canada, 2020*.; 2021. Accessed May 18, 2023. https://www150.statcan.gc.ca/n1/daily-quotidien/210531/dq210531d-eng.htm

93. Canadian Heritage. *Statistics on Official Languages in Canada*.; 2019. Accessed May 18, 2023. https://www.canada.ca/en/canadian-heritage/services/official-languages-bilingualism/publications/statistics.html

94. Haddad C, Sacre H, Zeenny RM, et al. Should samples be weighted to decrease selection bias in online surveys during the COVID-19 pandemic? Data from seven datasets. *BMC Med Res Methodol*. 2022;22(1):63. doi:10.1186/s12874-022-01547-3

95. Statistics Canada. *The Distribution of Temporary Foreign Workers across Industries in Canada*.; 2020. Accessed March 8, 2024. https://www150.statcan.gc.ca/n1/pub/45-28-0001/2020001/article/00028-eng.htm

# Additional File Information

Additional descriptives of predictor items and sleep variables, individual trajectory models, Model Selection data, Pearson’s correlation matrix, and full logistic regression models are provided in "Additional File A1.docx".
